# Metabolic rewiring and biomass redistribution enable optimized mixotrophic growth in Chlamydomonas

**DOI:** 10.1073/pnas.2522572123

**Published:** 2026-01-22

**Authors:** Somnath Koley, Kevin Foley, Zoee Perrine, Stewart A. Morley, Shrikaar Kambhampati, Olivia Gomez, Kevin L. Chu, Yi-Hsiang Chou, Michael Wei, Shin-Cheng Tzeng, Russell Williams, James G. Umen, Doug K. Allen

**Affiliations:** ^a^Donald Danforth Plant Science Center, St. Louis, MO 63132; ^b^University of Tennessee-Oak Ridge Innovation Institute, University of Tennessee, Knoxville, TN 37996

**Keywords:** metabolic flux analysis, central carbon metabolism, algae, glyoxylate cycle, isotopic labeling

## Abstract

Aquatic photosynthetic systems account for approximately one-half of all global carbon assimilation and could be a significant source of renewable fuels and feedstocks. However, rapid growth and biomass production in algae have not always translated into high product yields, partly because central metabolism is context specific, with metabolic fluxes being influenced by nutrient conditions and other environmental factors. In the green microalga *Chlamydomonas reinhardtii* (Chlamydomonas), mixotrophic cultures (acetate + light) grow far faster than phototrophic (light only) or heterotrophic (acetate + dark) cultures, even though acetate partially suppresses photosynthesis. Here, an isotopic dilution strategy with unlabeled acetate was combined with ^13^CO_2_ transient labeling to perform isotopically nonstationary metabolic flux analysis (INST-MFA) and to directly compare autotrophic and mixotrophic metabolism in Chlamydomonas supported by data from transcriptomics, proteomics, and metabolomics. INST-MFA indicated that acetate induces a synergistic rewiring of metabolism, conserving carbon by using the glyoxylate cycle and suppressing gluconeogenesis, the latter of which was discordant with omics results and prior models. Additionally, our data provide a plausible rationale for the well-known suppression of photosynthesis by acetate. We propose that reduced total protein content in mixotrophic versus phototrophic cells, much of which is attributed to reduced levels of photosynthetic proteins, decreases the costly metabolic burden of protein synthesis and represents a growth rate optimization strategy.

## Significance

Algae are a promising source of renewable feedstocks for energy, natural products, and biomass. Still, carbon partitioning strategies that dictate algae growth and biomass composition remain incompletely understood, limiting rational engineering efforts. Isotope-assisted metabolic flux analysis enables reconstruction of metabolic networks based on empirically deduced reaction rates. Using this approach, we compared photosynthetic and mixotrophic metabolism in the green alga Chlamydomonas and observed a metabolic synergy arising from acetate utilization in light. Acetate induced the glyoxylate cycle and, despite omics data and prior modeling predictions, suppressed gluconeogenesis while also reducing photosynthetic flux. Further, our flux maps illustrate how partial suppression of photosynthesis by acetate may help achieve growth optimization by reducing the costly burden of protein synthesis.

Aquatic photosystems account for half of all inorganic carbon assimilated globally and are a significant but largely untapped resource for food and biotechnology (1). Green microalgae can rapidly grow with CO_2_ assimilation rates 10 to 50 times higher than land plants. Their growth potential and ability for cultivation on land unsuitable for agriculture make them an attractive feedstock source for biofuels and other bioproducts (2). Many algal species have flexible metabolism and can assimilate and use available organic carbon, such as glucose or acetate, to support mixotrophic or heterotrophic growth. This flexibility can be exploited to improve the yield of specific metabolites or bioproducts such as triacylglycerol. Despite the strong growth-promoting influence of organic carbon in many microalgal species, there are little empirical data on the impact of organic carbon on algal metabolism.

*Chlamydomonas reinhardtii* (Chlamydomonas) is a haploid unicellular green alga with a sequenced (3) and extensively annotated genome of ~120 Mb (4) and is a model for understanding photosynthetic metabolism. Chlamydomonas can be cultured autotrophically (light), mixotrophically (light + acetate), or heterotrophically (dark + acetate), and grows most rapidly under mixotrophic conditions (5, 6). Responses of Chlamydomonas to changes in light or nutrient conditions have been studied by systems-level omics (7–12); however, levels of gene expression, metabolic enzymes, and metabolites are not accurate predictors of metabolic flux (13, 14). Mathematical modeling with flux balance analyses (FBA; all abbreviations are listed in Dataset S1) of Chlamydomonas or other algae (6, 15–17) has enabled the reconstruction of large metabolic networks and flux predictions for further testing; however, FBA cannot accurately estimate use of complementary pathways or account for enzyme reversibility, and is dependent on a presumed objective function such as maximizing growth rate, which is an oversimplification that does not adequately account for metabolic inefficiency and biomass turnover. Isotope-assisted metabolic flux analysis (MFA) infers flux values directly from measured labeling patterns (18–20) and produces a more comprehensive and quantitative assessment of metabolism than FBA. Indeed, existing MFA studies of algae under photo- or heterotrophic conditions (20–22) showed differences from FBA-based predictions because they did not conform to the principal assumption of FBA that metabolism is optimized for maximum growth or biomass production.

Flux maps derived from isotopic labeling have not directly compared metabolism for different trophic conditions within a single algal species under controlled growth conditions. Here, we used isotopically nonstationary MFA (INST-MFA) (23, 24) with ^13^CO_2_ labeling to compare autotrophic and mixotrophic metabolism of Chlamydomonas. The analyses quantitatively describe the metabolic flow of organic (acetate) and inorganic (CO_2_) carbon in steady-state photobioreactor cultures without external perturbation and were compared with transcriptomic and proteomic data to enable benchmarking of flux measurements against transcript and protein levels of metabolic enzymes. The results confirmed previous observations that in the presence of acetate, Chlamydomonas cells reduced their photosynthetic carbon assimilation rate and utilized carbon-conserving pathways, including a substantial glyoxylate bypass that circumvented TCA-based respiratory carbon loss. Along with reduced photosynthetic rates, mixotrophic cells also had significantly less total protein than phototrophic cells, enabling them to increase their growth rate while using less total energy than phototrophic cells. Under mixotrophy, acetyl-CoA was produced directly from acetate, thereby reducing the decarboxylation of pyruvate as the source of acetyl-CoA under autotrophic conditions. Prior FBA-based models predicted that acetate in mixotrophy would be converted to starch and sucrose through gluconeogenesis (6). Despite omics data showing the capacity for gluconeogenesis (presented here), INST-MFA established that mixotrophic metabolism occurs without any detectable gluconeogenic activity and identified phosphoenolpyruvate carboxykinase (PEPCK) as a central node for controlling gluconeogenic flux, presumably through posttranslational regulation. Similarly, pyruvate dehydrogenase (PDH) flux in mixotrophy was negligible compared to autotrophy; however, protein and transcript levels of PDH were higher in mixotrophy versus autotrophy. Our results correct previous descriptions of mixotrophic and autotrophic metabolism in Chlamydomonas and rationalize the paradoxical-seeming observation of reduced photosynthetic flux but enhanced growth in the presence of acetate. We hypothesize that in the presence of acetate, cells undergo a metabolic adaptation that balances the high cost of producing photosynthetic proteins with their benefits for energy production and carbon capture.

## Results and Discussion

### Transient ^13^CO_2_ Labeling Probes Metabolic Flux without Culture Perturbation.

**Fig. 1.**
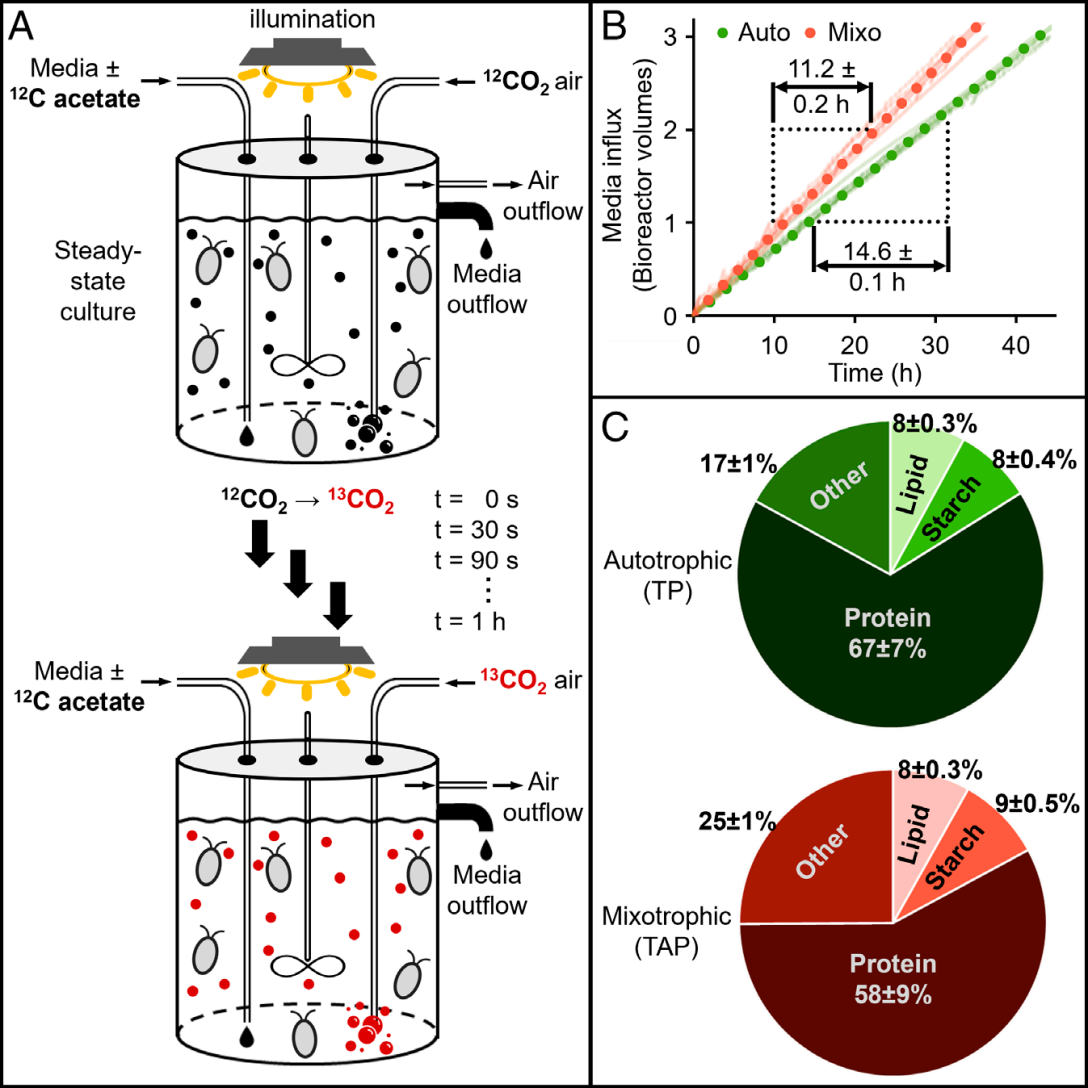
Experimental setup for isotope addition and growth metrics. (*A*) Schematic diagram of the turbidostat to visualize the changes during the isotopic study. (*B*) The volume of fresh media added was plotted against time to estimate turbidostatic growth rates. (*C*) Biomass composition of autotrophic and mixotrophic cells (mean ± SD; n = 5 for lipid, starch, and protein, and other (cell wall, other carbohydrates, and nucleic acids). Auto: autotroph; Mixo: mixotroph.

We established a photobioreactor culture system for our experiments with cells grown turbidostatically under constant illumination. This setup creates a stable, steady-state environment that significantly increases reproducibility across replicate experiments compared with flask cultures that are subject to many additional sources of variability. Mixotrophic cultures grown in standard Tris-acetate-phosphate (TAP) media and autotrophic cultures grown in Tris-phosphate (TP) media were bubbled with air and maintained at the same optical density to ensure similar levels of illumination and nutrients (other than acetate) for each condition (Fig. 1*A*). As expected, mass doubling time in mixotrophy (11.1 h) was lower than in autotrophy (14.6 h) with corresponding specific growth rates of 0.062 h^−1^ (mixotrophy) and 0.048 h^−1^ (autotrophy) (Fig. 1*B*) (5, 25).

Carbon distribution within cells can change significantly under different growth conditions. To accurately constrain our metabolic models, we directly measured the distribution of carbon across significant biomass categories, including protein, starch, and lipids, and estimated others (cell wall, other carbohydrates, and nucleic acids) in cultures grown under each condition (Fig. 1*C* and *Materials and Methods*). Our data indicated that in autotrophic versus mixotrophic conditions, there was a higher proportion of protein (67% versus 58% by dry weight, respectively; *P* = 0.11) but similar amounts of lipid and starch, with the remainder distributed among other macromolecules, including cell wall and nucleic acids. To confirm that the difference in total protein between growth conditions was not due to sample variance or tied to overall growth rates, we repeated our measurements under similar conditions and included an autotrophic culture supplemented with 1% CO_2_ which grew at an intermediate rate between the mixotrophic culture with air and autotrophic culture with air (*SI Appendix*, Fig. S1). As in the earlier experiment, protein levels under mixotrophy (58%) were significantly lower than the autotrophic cultures (63%) (*P* = 0.0026) or the CO_2_-supplemented cultures (66%; *P* = 0.0001), and were not tied to relative growth rates (*SI Appendix*, Fig. S1). Among the macromolecular constituents, protein synthesis requires much more ATP than other biosynthetic processes (i.e., ATP consumed per unit biomass) (26–28), and its reduced representation in mixotrophic biomass suggests a decreased metabolic burden for cell growth in the presence of acetate.

**Fig. 2.**
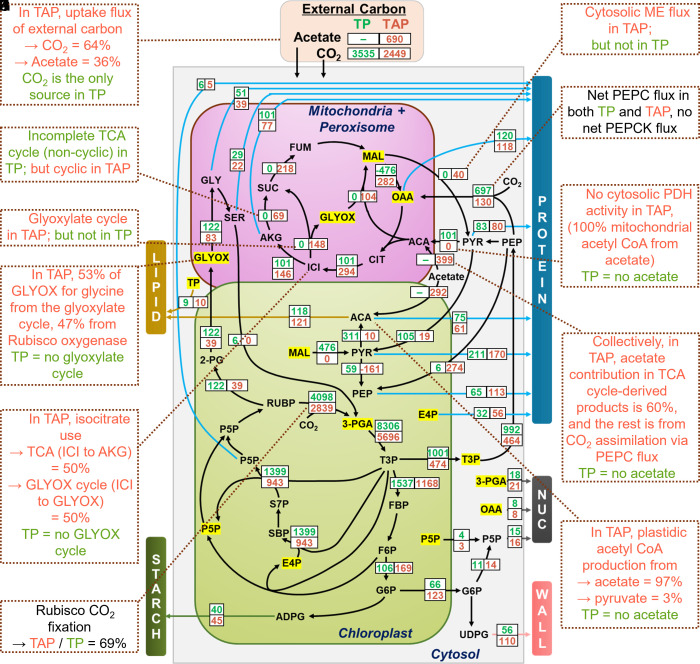
Autotrophic and mixotrophic carbon fluxes measured by INCA. Net flux values determined from autotrophic (TP, green) and mixotrophic (TAP, red) conditions are the medians of the 95% flux CI. They are presented after rescaling to adjust for the difference in doubling time between TP and TAP (Dataset S8). Flux values in µmol*g^−1^(DW)*h^−1^ before rescaling are presented in *SI Appendix*, Fig. S4. Metabolites highlighted in yellow are shown multiple times in different locations on this figure to reduce complexity, but represent single pools in the modeled flux analysis. The breakdown of protein and starch was included in the flux analysis (Dataset S8) but is not presented here to simplify the flux map. All abbreviations used are listed in Dataset S1. Callouts (*A*–*K*) describe key data and conclusions based on the flux map (calculations in Dataset S8). Green/red text in callouts signifies autotrophic/mixotrophic conditions, respectively.

For isotopic labeling, we used a synthetic air mixture with ^13^CO_2_ (350 ppm) in combination with N_2_ (78%) and O_2_ (21%) (Fig. 1*A*). This mixture can be rapidly and quantitatively equilibrated into the growth media without perturbing cultures. We did not use labeled acetate as its introduction would require either a complete replacement of growth media or a substantial change in acetate concentration, either of which would likely push cultures out of steady-state. Importantly, the metabolic flux of unlabeled carbon from acetate can still be measured indirectly in these experiments using ^13^C flux measurements and principles of mass conservation. The ^13^C enrichment was monitored by rapidly collecting biomass samples from cultures between 30 s and 60 min after isotope introduction. Differences in ^13^CO_2_ labeling between autotrophic and mixotrophic cultures reflected a combination of effects, including i) dilution of ^13^CO_2_ by unlabeled carbon from acetate; ii) faster growth and reduced photosynthetic carbon assimilation in mixotrophy versus autotrophy; and iii) reincorporation of unlabeled CO_2_ released from catabolism of acetate. Prior descriptions of Chlamydomonas metabolism (29) were used to develop the flux model network (Dataset S2) and were overlaid with proteomic and transcriptomic data (Datasets S3 and S4). For example, the transport of photoassimilates from the chloroplast to the cytosol via triose phosphate export (30), and hexose phosphate export for UDP-glucose production (29) were included in the flux model. Reactions to produce hexose phosphates from triose phosphates in the cytosol were excluded due to i) absence of transcripts for the corresponding enzymes, ii) similar labeling between phosphorylated hexoses and nucleotide sugars (Text S1, *SI Appendix*, Figs. S2 and S3), and iii) lack of cytosolic flux suggested by other algal studies (31). Biomass composition, growth rate, and isotope labeling data (Datasets S5–S7) were inputs for INST-MFA using the INCA platform (32). Flux values in both maps were initially measured on an absolute scale, i.e., mol flux per unit mass per unit time (*SI Appendix*, Fig. S4), but were later rescaled to account for the different growth rates between autotrophic and mixotrophic cultures (Fig. 2). This adjustment facilitated direct comparisons of flux partitioning between the two growth conditions.

### Acetate Rewires the TCA Cycle through the Glyoxylate Shunt but Does Not Activate Gluconeogenesis.

**Fig. 3.**
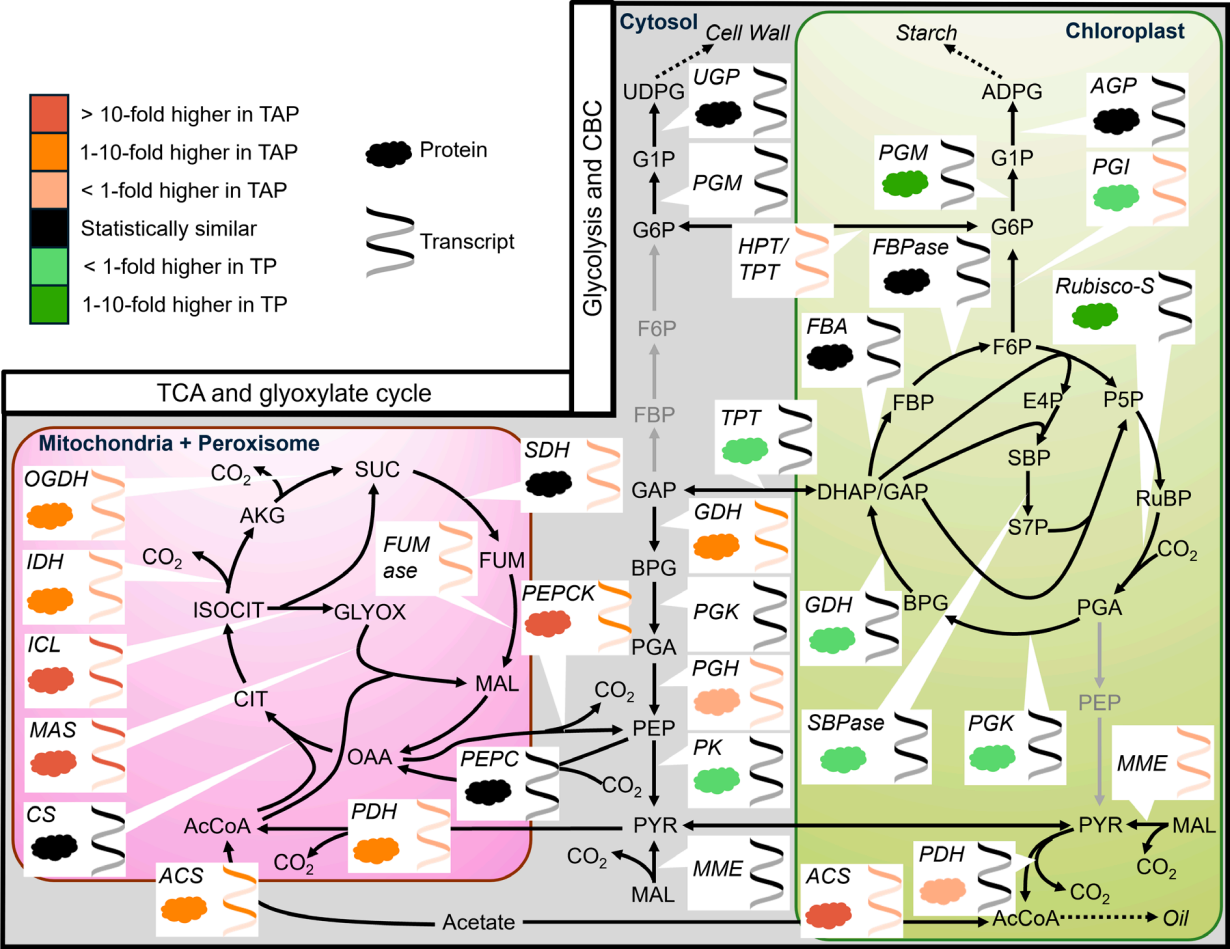
Transcriptomics and proteomics differences in CBC, glycolysis, TCA, and glyoxylate cycle between autotrophic and mixotrophic Chlamydomonas. When multiple paralogs of an enzyme are predicted to be in the same compartment, only data for the paralog with the highest expression are shown (Datasets S3 and S4). The net direction of flux is presented for bidirectional reactions using arrows. Gray arrows indicate low or absent transcript levels (*SI Appendix*, Fig. S3). In cases where protein data are unavailable, the corresponding symbol is omitted. Differential expression analysis was performed at a 95% confidence level, with fold difference ranges in the key (*Upper* *Left*). n = 3 for proteomics and n = 2 for transcriptomics.

One of the most striking contrasts between auto- and mixotrophic metabolism involved the flow of carbon through organic acids to produce amino acids and ATP. In mixotrophy, glutamate and aspartate family amino acids were made using a combination of a complete TCA cycle alongside the glyoxylate cycle (Fig. 2 *A* and *B* and Dataset S8). By contrast, in autotrophic conditions, the TCA pathway was noncyclic. Phosphoenolpyruvate (PEP), derived from photosynthesis, was converted to oxaloacetate (OAA), the precursor for aspartate-derived amino acids, or converted to pyruvate and acetyl-CoA, which condenses with oxaloacetate to form citrate. Citrate is subsequently converted to α-ketoglutarate and glutamate family amino acids, but unlike metabolism in mixotrophy, there was no detectable flux from α-ketoglutarate to succinate in autotrophy, which is needed to complete the TCA cycle (Fig. 2*A*). Consequently, in autotrophy, ATP is produced primarily through photosynthetic light reactions and not through oxidative phosphorylation in mitochondria, similar to the metabolism of autotrophic leaves (18, 33). Compared to autotrophy, mixotrophic cultures produced 29% more ATP through the TCA cycle (Dataset S9), with some of the additional ATP required to sustain the conversion of acetate to acetyl-CoA. The greater mitochondrial flux under mixotrophy versus autotrophy was consistent with multiomics data showing increased transcripts and proteins for nearly all TCA enzymes (Fig. 3) and increased pool sizes of succinate, malate, and fumarate in mixotrophic cells (*SI Appendix*, Fig. S5).

**Fig. 4.**
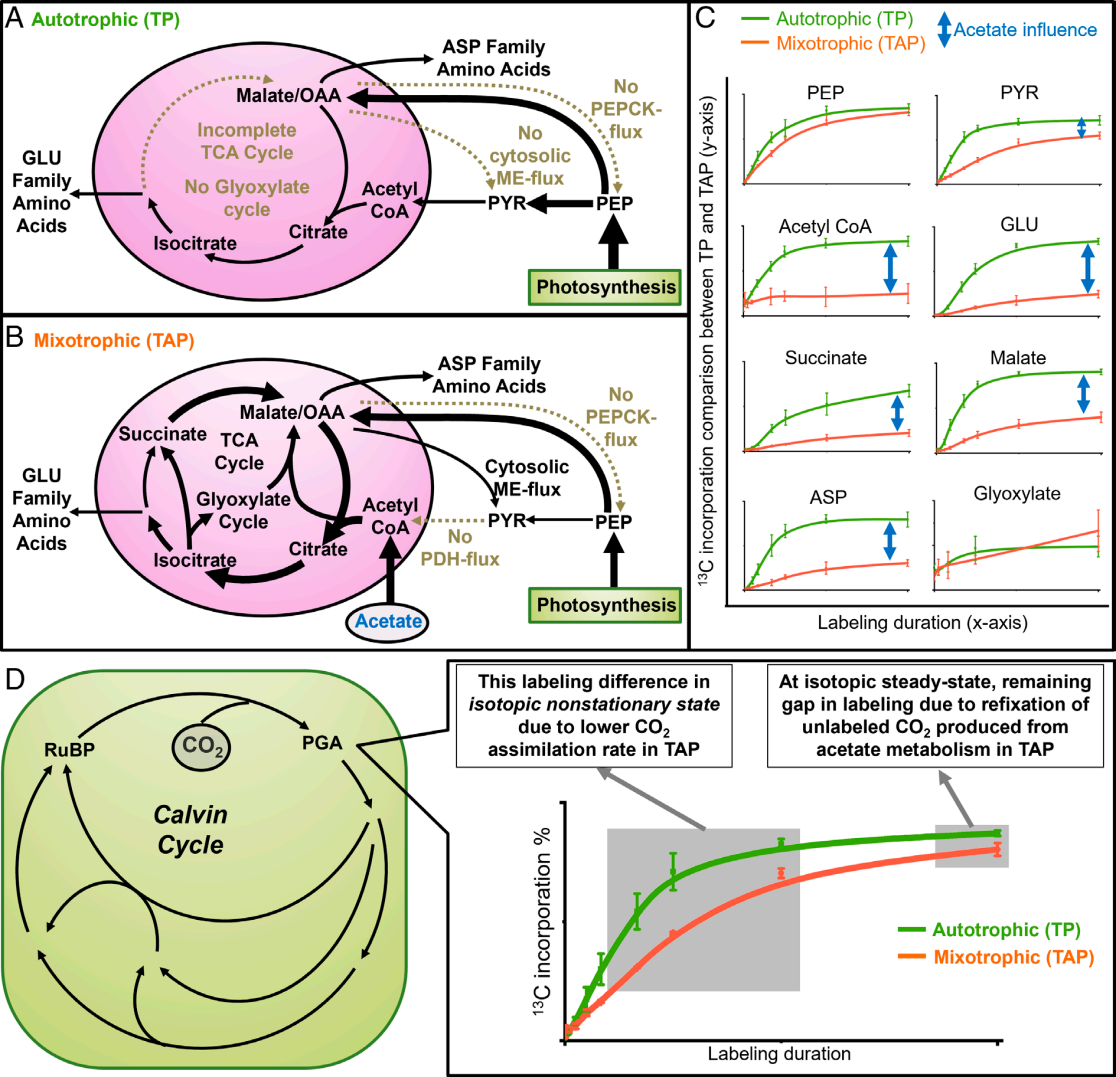
Differences between autotrophic and mixotrophic conditions in labeling of TCA and CBC metabolites. Summary of (*A*) autotrophic and (*B*) mixotrophic metabolism supported by the (*C*) differences in labeling of central carbon metabolites. Arrow thickness in (*A* and *B*) represents flux intensity- thicker arrows indicate higher flux, while thinner arrows indicate lower flux. (*D*) Labeling differences in phosphoglycerate (the immediate product of photosynthetic CO_2_ assimilation) between autotrophs and mixotrophs during the approach to steady-state. PEP: phosphoenolpyruvate; PYR: pyruvate; GLU: glutamate; ASP: aspartate; TCA: tricarboxylic acid; PGA: phosphoglycerate; RuBP: Ribulose bisphosphate. Mean ± SD; n = 4 for autotrophy and n = 6 for mixotrophy. y-axes are scaled linearly but not labeled since they are meant to show relative differences between autotrophic and mixotrophic labeling rates. Labeling details can be found in Dataset S7.

A deeper inspection of labeling data (Fig. 4 *A*–*C* and *SI Appendix*, Fig. S6) provided further insight into how acetate-derived carbon is partitioned under mixotrophy, with 60% going to TCA-derived products including amino acids (Fig. 2*C*). Glutamate, an amino acid derived from the α-ketoglutarate node of the TCA cycle, was 59% less labeled in mixotrophy than autotrophy at 1 h, indicating that the unlabeled acetate provided most of the carbon for biosynthesis of α-ketoglutarate-derived amino acids (glutamate, glutamine, proline, and arginine). This was also reflected by the complete absence of M+4 and M+5 glutamate isotopologues in mixotrophy versus autotrophy due to ^12^C from unlabeled acetate (“M” implies molecular weight of unlabeled compound; *SI Appendix*, Fig. S7). Acetate also reduced oxaloacetate-derived amino acid labeling (aspartate, asparagine, methionine, threonine, and lysine), but less than for the α-ketoglutarate node, 49% (Fig. 4*C*). The difference in labeling for the oxaloacetate-derived amino acids compared with glutamate-derived amino acids is from the contribution of ^13^C from active phosphoenolpyruvate carboxylase (PEPC) flux to the oxaloacetate pool via glycolysis, resulting in significant M+3 and M+4 isotopologues in four-carbon aspartate (*SI Appendix*, Fig. S7).

In autotrophy, the glyoxylate cycle was inactive. But under mixotrophy, 50% of isocitrate was partitioned through the glyoxylate shunt (Fig. 2*D*), thus bypassing carbon-respiring steps in the TCA cycle. Consequently, under mixotrophy, the glyoxylate cycle contributed 68% and 78% of the carbon in succinate and malate, respectively, both of which shared intermediates from concurrent cyclic TCA and glyoxylate cycle operation (Dataset S8: relative flux calculation). This metabolic rewiring under mixotrophy was detectable by the reduction in metabolite labeling relative to autotrophy, even though a significant fraction of the glyoxylate cycle carbon came from unlabeled acetate (Fig. 4*C*). The glyoxylate shunt is a carbon-conserving mechanism that often sustains gluconeogenesis. Concordantly, omics data indicated higher transcript and protein abundance for signature enzymes of the glyoxylate shunt (malate synthase and isocitrate lyase) in mixotrophy compared with autotrophy (Fig. 3 and *SI Appendix*, Figs. S8 and S9). These data were consistent with previous findings that Chlamydomonas isocitrate lyase mutants, which impair the glyoxylate cycle, had reduced growth compared with wild-type cells in mixotrophic but not autotrophic conditions (34).

The glyoxylate cycle can supply carbon for gluconeogenesis in Chlamydomonas (6), and omics data presented here indicate higher expression and abundance of the signature gluconeogenesis enzyme, PEPCK, in mixotrophy relative to autotrophy (Fig. 3 and *SI Appendix*, Fig. S9). Surprisingly, gluconeogenic flux was not detected under either trophic condition, as indicated by isotopic labeling and the corresponding metabolic flux maps. Under both conditions, the net flux was from phosphoenolpyruvate to oxaloacetate (i.e., PEPC flux) (Fig. 2*E*). The lack of flux in the opposite direction (i.e., PEPCK flux supporting gluconeogenesis) was corroborated by the similarity in labeling of glycolytic intermediates in mixotrophy and autotrophy (*SI Appendix*, Fig. S6). For example, phosphoenolpyruvate, which is the immediate product of PEPCK activity, and phosphoglycerate, which is the immediate product of ribulose-1,5-bisphosphate carboxylase/oxygenase (Rubisco) activity in the Calvin-Benson cycle (CBC), were labeled similarly between conditions by the 1 h time point when labeling approached isotopic equilibrium. This labeling pattern indicated phosphoenolpyruvate is produced from CBC-derived phosphoglycerate, rather than via PEPCK activity. Additionally, the lack of significant isotope dilution of other glycolytic sugar phosphates in mixotrophy versus autotrophy indicated that unlabeled acetate-derived carbon did not contribute significantly to glycolytic or CBC intermediates through gluconeogenesis. Further confirmation was found in the labeling of organic acids in mixotrophy. ^13^C-PEP-supplied carbon was converted to oxaloacetate through incorporation of ^13^CO_2_ by the PEPC reaction, which resulted in M+4 malate. If malate were derived principally from succinate, the labeling profile would be similar to that of succinate, which was capped at M+2 (*SI Appendix*, Fig. S10). Taken together, these results suggest that despite maintaining significant levels of PEPCK enzyme, it is not utilized in central carbon metabolism under either of our tested trophic conditions, though presumably it would be needed for strict heterotrophic metabolism to produce hexose and may also be active in diurnal cultures during the dark phase when cells cannot perform photosynthesis. We speculate that in the presence of light PEPCK is held inactive through posttranslational changes, possibly through light-dependent phosphorylation/dephosphorylation or oligomerization (35, 36). The MFA-based results underscore the potential pitfall of relying on transcript or protein abundance as proxies for metabolic rates in modeling studies (14, 37).

NADP-dependent malic enzyme 2 (MME2) is one of the most abundant malic enzyme isoforms in Chlamydomonas and converts malate to pyruvate in the cytosol. The flux map indicated cytosolic malic enzyme activity in mixotrophy but not autotrophy (Fig. 2*F*). Consequently, in contrast to other glycolytic metabolites that did not undergo significant label dilution in the presence of acetate, mixotrophically derived pyruvate was less labeled relative to autotrophic pyruvate (Fig. 4*C* and *SI Appendix*, Fig. S6). This labeling difference indicated the transport of TCA-derived mitochondrial malate into the cytosol, where it was converted to pyruvate by malic enzyme. In contrast, autotrophic cells had no cytosolic malic enzyme activity (Fig. 2*F*) but converted malate to pyruvate in the chloroplast, catalyzed by MME3 (Fig. 3).

### Noncanonical Use of the Glyoxylate Cycle to Produce Glycine and Serine.

Although photorespiration and glycolate production were low in Chlamydomonas due to its efficient carbon concentrating mechanism (i.e., ~1 to 3% of carboxylation; Fig. 2) (38) they were the primary route of serine and glycine synthesis in autotrophic Chlamydomonas cells, just as in photosynthetic leaves of land plants. However, under mixotrophy nearly one-half of the glyoxylate-carbon used in serine and glycine biosynthesis was derived from a noncanonical source of glyoxylate originating in the glyoxylate cycle, with the remainder derived from Rubisco oxygenation (Fig. 2*K*). Consequently, the extent of glycine labeling in mixotrophy fell in between that of 2-phosphoglycolate, a precursor exclusively from Rubisco oxygenase activity (*SI Appendix*, Fig. S13), and glyoxylate, which was derived from both Rubisco oxygenation and from the glyoxylate cycle. Consistent with this observation, under mixotrophy (versus autotrophy), we observed reduced protein levels of glycolate dehydrogenase that produces glyoxylate from photorespiration, and increased levels of glyoxylate cycle enzymes that convert glyoxylate to glycine and serine (*SI Appendix*, Fig. S14). Metabolic rewiring to include glyoxylate production from the glyoxylate cycle has been postulated in lipid-producing transgenic leaves to inhibit photosynthetic carbon assimilation, but whether it displaced photorespiratory production of glycine or serine was not determined (39). Further, the flux analysis of Chlamydomonas under autotrophic and mixotrophic conditions indicated that glycine and serine were not recycled to PGA as occurs in a closed photorespiratory cycle (Fig. 2) but instead contributed almost exclusively to protein synthesis. A recent study (40), showed that Chlamydomonas actively excretes glycolate under photorespiratory stress, demonstrating that carbon can leave the pathway rather than being converted back to glycerate as in the canonical closed photorespiratory pathway. Loss of the chloroplast-envelope transporter LCI20 interrupted the glyoxylate-to-glycine conversion with a reduction in glycolate dehydrogenase abundance. These changes diverted carbon away from the closed photorespiration cycle and resulted in metabolite leakage. Even with an active carbon-concentrating mechanism (CCM), photorespiration persists and channels glycine and serine into nitrogen assimilation and protein biosynthesis, providing evidence for distinct pathway operation. Such open photorespiratory loops as we observed in Chlamydomonas can enhance net carbon assimilation (41) and have also been reported in leaves (42).

### Mixotrophic Cells Conserve Carbon through Reduced Decarboxylation and Use Acetate for Anabolic Metabolism.

Acetyl-CoA produced directly from acetate under mixotrophy does not require a decarboxylation step, but under autotrophy, acetyl-CoA is derived from pyruvate through PDH, resulting in the loss of one carbon. The flux map indicated that 97% and 100% of acetyl-CoA in the chloroplast and mitochondria, respectively, was derived directly from acetate under mixotrophy (Fig. 2 *G* and *H*), thus improving carbon use efficiency by nearly 33% in this step. Mechanisms that avoid or minimize decarboxylation, such as the glyoxylate cycle or through restricting PDH flux, reduce the total carbon loss. As a result, mixotrophic respiration was 14% of total biomass compared to 36% in autotrophy (Dataset S8: relative flux calculation). This result was counterintuitive because the supply of acetate and cyclic TCA might imply that respiration would be high, but on the contrary, mixotrophic metabolism was carbon-conserving due to rewiring of the TCA and glyoxylate cycles. Multiple isoforms of acetyl-CoA synthetase which converts acetate to acetyl-CoA were expressed and abundant in mixotrophic cells (Fig. 3). Under mixotrophic conditions, the inferred import of acetate to the chloroplast and its conversion to acetyl-CoA was validated by inspecting the isotopic signatures in branched-chain amino acids synthesized in the chloroplast. The absence of the M+6 and M+5 isotopologues of six-carbon leucine (*SI Appendix*, Fig. S7) in mixotrophic conditions was a direct result of unlabeled, acetate-derived acetyl-CoA being used as a precursor for leucine synthesis (Fig. 2*H*). In contrast, under autotrophic conditions, total PDH flux was 40-fold higher (Fig. 2) and pyruvate abundance eightfold higher than in mixotrophic conditions to allow sufficient production of acetyl-CoA in the absence of external acetate (*SI Appendix*, Fig. S5). Similar to the discordance between flux and omics data for PEPCK, the reduced PDH flux in mixotrophic conditions was not reflected by a reduction of cytosolic or chloroplast-localized PDH proteins or transcripts, presumably because PDH is also needed under dark conditions like PEPCK (Fig. 3).

### Autotrophic Cells Have Elevated Rates of Protein Synthesis and Turnover.

In addition to predicting flux through anabolic metabolism to generate biomass, the flux maps established the rates of biomass turnover which are not accounted for in FBA analyses or previously reported MFAs in Chlamydomonas; although protein and starch turnover have been described in plants (18, 43–45) and algae exposed to elevated light levels (12). Protein synthesis rates and turnover rates were significantly elevated under autotrophy with 53% of synthesized protein turned over versus 32% in mixotrophy (Dataset S8). The balance between synthesis and turnover establishes the net protein synthesis rates which are higher in autotrophy than in mixotrophy. A deeper inspection of the labeling results provided further insight. Near the end of the labeling experiment, amino acids remained incompletely labeled in both autotrophic and mixotrophic conditions, indicating that additional sources of unlabeled carbon were contributing to these pools. Although in mixotrophy dilution of ^13^C labeling in amino acids can be due to turnover of preexisting unlabeled proteins as well as the presence of unlabeled acetate in the system, in autotrophy, incomplete labeling is the sole result of turnover of unlabeled protein, resulting in an unlabeled amino acid contribution to total pools. Thus, unlabeled (i.e., M+0 isotopologue) amino acids (Dataset S7) present at the end of the labeling period in autotrophy indicated protein turnover. Elevated protein synthesis and turnover per gram biomass in autotrophy therefore imposes a greater energetic burden on cells than in mixotrophy where less total protein is synthesized to create and maintain protein biomass. It is unclear why autotrophic cells have higher protein turnover rates, but this may be linked to greater photodamage as autotrophic cells have higher overall levels of photosynthesis and photosynthetic proteins than mixotrophic cells (see also next section).

The differences in flux ratios between starch breakdown and synthesis were modestly higher under mixotrophy (40%) versus autotrophy (33%) (Dataset S8). When acetate was present, the total starch content increased slightly (Fig. 1*C*). Elevated starch synthesis flux (mixotrophy to autotrophy ratio of 1.7) was consistent with higher levels of starch biosynthetic enzymes under mixotrophy (STA2 and SBE3; Dataset S3); however, starch breakdown flux was also increased in mixotrophy versus autotrophy (Dataset S8), consistent with upregulation of the starch catabolic enzyme (PHO3; Dataset S3). The increased starch breakdown rate under mixotrophy contributed to an eightfold larger glucose pool than in autotrophy (*SI Appendix*, Fig. S5).

In our model, acetyl-CoA from lipid catabolism could not be distinguished from external acetate and was omitted; however, we observed unlabeled acetyl-CoA (M+0; 11%) in autotrophic cells near the end of the labeling period (Dataset S7), suggesting some contribution of lipid turnover to acetate pools (46).

### Mixotrophic Cells May Optimize Growth by Suppressing Photosynthesis.

Consistent with prior observations (47), the presence of acetate reduced ^13^CO_2_ assimilation and CBC flux by 31% (Fig. 2*I*) but supported faster growth. Our flux analysis suggested a 24 to 34% higher rate of total carbon incorporation in mixotrophy versus autotrophy (*SI Appendix*, Fig. S4 and Dataset S8), commensurate with a 30% higher growth rate of TAP-grown cells versus TP-grown cells (Fig. 1*B*). In total, 36% of mixotrophic biomass was derived from acetate (Fig. 2*J*). The reduced photosynthesis caused by acetate was reflected in slower ^13^C incorporation rates at early labeling times (Fig. 4*D*). When an isotopic steady state was nearly reached at 1 h into the labeling period, phosphorylated intermediates and amino acids proximal to the CBC exhibited comparable ^13^C enrichment in autotrophic versus mixotrophic conditions (*SI Appendix*, Fig. S6). Thus, acetate-derived carbon contributed minimally to metabolite pools associated with the CBC and photosynthetic metabolism through reassimilation of unlabeled CO_2_ released by respiration of acetate (Fig. 4*D*). We also estimated the individual labeling of all three carbon atoms in plastidic 3-phosphoglycerate at isotopic steady state and found that mixotrophic cells were labeled 5% less than autotrophic cells (*SI Appendix*, Fig. S11). The decreased labeling in mixotrophy is attributed to fixation of unlabeled acetate-derived CO_2_ from respiration, indicating the proportion of CO_2_ derived from unlabeled acetate

At face value, the suppression of photosynthesis by acetate seems paradoxical: Cells appear to be forgoing untapped growth potential from unused photosynthetic capacity. However, protein synthesis is an energetically-costly biosynthetic process in cells, and a quantitative estimate of major photosynthetic protein complexes in Chlamydomonas cells found that they accounted for approximately 14% of total cellular protein (measured under mixotrophy) (48). Moreover, this estimate did not include CBC proteins (besides Rubisco), light-harvesting complexes, and many other ancillary proteins that support photosynthesis. We observed that the relative proportion of protein to total biomass was significantly decreased under mixotrophy compared to autotrophy (Fig. 1*C* and *SI Appendix*, Fig. S1), with lower abundance of proteins involved in photosynthesis, and photosynthetic metabolism measured with proteomics (Fig. 3 and *SI Appendix*, Fig. S12). For example, subunits of Rubisco and sedoheptulose 1,7-bisphosphatase, signature proteins in the CBC, were significantly less abundant in mixotrophic cells than in autotrophic cells (1.5 to 3.7-fold lower; Fig. 3 and Datasets S3 and S4). Interestingly, the transcript levels of genes encoding the above proteins were similar under both conditions, suggesting that their protein abundances may be controlled posttranscriptionally. Additionally, the CCM is energetically expensive to construct and maintain, involving numerous proteins that form the pyrenoid matrix (49), and requiring ATP to drive bicarbonate transport and concentration processes (50, 51). Acetate metabolism leads to a reduced investment in the CCM, as reflected by decreased abundances of associated proteins (*SI Appendix*, Fig. S12).

To assess whether carbon limitation or acetate-dependent metabolic rewiring determines protein content, we grew PBR cultures with excess CO_2_ (1% CO_2_) under autotrophic conditions along with standard autotrophic and mixotrophic cultures supplied with air. Relative to mixotrophy, the autotrophic conditions contained more protein regardless of carbon limitation or sufficiency, indicating the effect we observed with acetate was not related to growth rate or carbon limitation (*SI Appendix*, Fig. S1). Based on these observations, we propose that reduced photosynthesis in the presence of acetate does not represent a wasted opportunity but is instead a metabolic adaptation that enables faster growth by balancing the benefit of photosynthesis against its cost of maintenance. In other words, mixotrophy decreases the need for photosynthesis because the modified metabolism when acetate is available creates a new cost–benefit set point for photosynthetic capacity. The effects of acetate addition on photosynthesis occur within minutes and include downregulation of PSII, evidenced by decreased fluorescence yield (Fm’) (52) and may result from direct inhibition of PSII by acetate (53). This rapid shift caused by acetate may induce signaling, which subsequently downregulates the steady-state production of photosynthetic proteins.

In summary, prior descriptions of mixotrophic metabolism in Chlamydomonas assumed a significant flow of acetate-derived carbon into gluconeogenesis to produce starch and sucrose, essentially describing a merger of heterotrophic dark metabolism and autotrophic light metabolism, each running in parallel. Instead, we found mixotrophic growth in Chlamydomonas is driven by a metabolic rewiring that partially separates light-driven reactions and hexose metabolism from organic acid metabolism and amino acid synthesis. The latter is propelled extensively by acetate-derived acetyl-CoA entering the TCA and glyoxylate cycles, while hexose metabolism is almost exclusively driven by CO_2_ entering the CBB cycle. This metabolic subspecialization is likely to be advantageous, as it minimizes carbon loss by bypassing the TCA cycle decarboxylation steps and instead exploits the glyoxylate cycle, thereby eliminating the energetically costly conversion of acetate-derived carbon to hexose through gluconeogenesis. The synergy created by this subspecialization is likely to be responsible for the paradoxical observation of faster growth in mixotrophic cultures (light + acetate) than the sum of growth rates of cells using autotrophic (light) plus heterotrophic (dark + acetate) metabolism. Thus, suppression of photosynthesis by acetate appears to be a metabolic adaptation that balances the benefit of maintaining photosynthetic protein machinery versus its substantial metabolic production cost of energy and carbon. Our data provide a framework for future modeling studies and experimental work aimed at further testing this idea.

## Materials and Methods

Method details for culturing, sample extraction, omics analysis, and biomass measurement are described in *SI Appendix*, *Supplementary Materials and Methods*.

### ^13^CO_2_ Isotopic Labeling Experiments.

Isotopic labeling experiments were carried out with ^13^CO_2_-enriched synthetic air (^13^CO_2_/N_2_/O_2_ ratio of 0.033:78:21.967; Aldrich, Milwaukee, WI) starting at a high flow rate to exhaust the existing unlabeled air and reduced to 250 mL/min for the duration of the experiment. Samples of labeled biomass were harvested at 0 s, 30 s, 90 s, 3 min, 5 min, 10 min, 15 min, 30 min, and 1 h by rapid filtering and collection of biomass that was flash frozen in liquid nitrogen within several seconds after collecting under equivalent light conditions. Approximately 25 mL of culture was fast-filtered (2.0 µm pore size, Merck Millipore, Cork, IRL) through a vacuum filtration apparatus. Frozen filters were stored at −80 °C until extracted as described previously (54, 55). Sample extraction and measuring metabolites for flux analysis are detailed in *SI Appendix*, *Supplementary Materials and Methods*.

### MFA.

INST-MFA was performed using the INCA package (32) on a MATLAB-based distributed computing platform and standalone computers. An algal metabolic network (Dataset S2) was developed using the Chlamydomonas Sourcebook (56), *C. reinhardtii* genome (3), standard textbook descriptions of central metabolism, other ref. 29, and based on the combination of transcriptomic and proteomic data from this study. In addition to the network, models included measured fluxes to biomass components, tracer descriptions, and isotopic labeling measurements obtained through mass spectrometry (Datasets S5–S7). The resulting flux maps were the consequence of fitting ordinary differential equations throughout the iterative minimization process to get a best-fit solution from randomized initial starting points within the flux solution space. CI were estimated through the parameter continuation process available in the software.

## Supplementary Material

Appendix 01 (PDF)

Dataset S01 (XLSX)

Dataset S02 (XLSX)

Dataset S03 (XLSX)

Dataset S04 (XLSX)

Dataset S05 (XLSX)

Dataset S06 (XLSX)

Dataset S07 (XLSX)

Dataset S08 (XLSX)

Dataset S09 (XLSX)

## Data Availability

Mass spectrometry data have been deposited in Zenodo (DOI: 10.5281/zenodo.16421485 and 10.5281/zenodo.16421550) (57, 58).
