# Supplementary material for: Metabolic rewiring and biomass redistribution enable optimized mixotrophic growth in Chlamydomonas
Source: Proc Natl Acad Sci U S A. 2026 Jan 22;123(4):e2522572123. doi: 10.1073/pnas.2522572123 (PMC12846818; doi:10.1073/pnas.2522572123)
Supplement: Supplementary file 1 — Appendix 01 (PDF) [file pnas.2522572123.sapp.pdf]

PNAS

Supporting Information for

Metabolic rewiring and biomass redistribution enable optimized mixotrophic growth in Chlamydomonas

Somnath Koley, Kevin Foley, Zoee Perrine, Stewart M. Morley, Shrikaar Kambhampati, Olivia Gomez, Kevin L. Chu, Yi-Hsiang Chou, Michael Wei, Shin-Cheng Tzeng, Russell Williams, James G. Umen, Doug K. Allen

Corresponding authors:
Doug K. Allen (dallen@danforthcenter.org), James G. Umen (jumen@danforthcenter.org), Somnath Koley (skoley@utk.edu)

This PDF file includes:

- Supporting text
- Supplementary Materials and Methods
- Figures S1 to S14
- Legends for Datasets S1 to S9
- SI References

Other supporting materials for this manuscript include the following:

- Datasets S1 to S9

Supporting Information Text

Text S1: Transport of hexose phosphates from chloroplasts to cytosols for UDP-glucose production:

Subcellular compartmentalization and protein targeting allow equivalent biochemical steps to be present in more than one organelle with different fluxes in each compartment (1, 2). In vascular plants, ADP-glucose, a precursor to starch, is produced from CBC-derived hexose phosphates inside chloroplasts. On the other hand, the formation of UDP-glucose, which is crucial for carbohydrate and cell wall biosynthesis, involves the transport of triose phosphates from the chloroplast to the cytosol where they are converted to hexose phosphate precursors (3–5). Consequently, the labeling in UDP-glucose is significantly different than ADP-glucose in plants (4). In Chlamydomonas, hexose phosphate, not triose phosphates, are thought to be exported from the chloroplast to the cytosol for UDP-glucose production (6). Unlike the case in plants, we observed similar labeling between plastidic ADP-glucose and cytosolic UDP-glucose in autotrophy and mixotrophy, which implied a rapidly equilibrating precursor pool for nucleotide sugar synthesis between the two compartments (Figure S1). This metabolite exchange may be mediated by predicted chloroplast-localized hexose phosphate translocators [HPT1, HPT2 and TPT25; (7, 8)] (Dataset S4, Figure S2). The results were also supported by insubstantial cytosolic localized paralogs for some glycolytic enzymes, suggesting the need for direct transport of hexose phosphates from chloroplasts. For example, transcripts for cytosol-targeted fructose bisphosphate aldolase and fructose 1,6-bisphosphatase were present at non-significant levels and expression of other genes that convert glycolytic intermediates, such as glucose-6-phosphate isomerase, were detected only for those with chloroplast targeting (Figure S2, Dataset S3-S4). These observations are consistent with *in vitro* subcellular fractionation activity assays (22) that indicate hexose phosphates are interconverted in the chloroplast and exported for cell wall production.

Supplementary Materials and Methods:

Algae culturing and bioreactor growth:

Plates: *Chlamydomonas reinhardtii* strains 21GR mt (+) (CC1690) was obtained from the Chlamydomonas Resource Center (https://www.chlamycollection.org) and was maintained on tris acetate phosphate (TAP) agar plates (TRIS- 2 M, 0.1 acetic acid, pH 7.4) at room temperature. Shake flask starter cultures (500 mL Erlenmeyer flasks with 300 mL liquid TAP media) were inoculated from plates and maintained at 25°C, 100 μmol/m^{2}s light intensity (58% red light/ 42% blue light), with filtered air through a submerged glass pipette.

Bioreactors: Cultures were grown at constant cell density in environmental photobioreactors (500 mL; Phenometrics™, East Lansing, MI) in either Tris-acetate-phosphate (TAP), pH 7.4 or Tris-phosphate (TP), pH 7.4 media (9). TP was prepared by omitting acetic acid and substituting Tris-base with Tris-HCl pH 7.4. Sterile tris phosphate (TP) media (1M tris, pH 7.4) or containing acetate (TAP; acetate 17mM) were supplied to reactor cuvettes operating turbidostatically to ensure a metabolic steady-state. An OD 750 of 0.6 (250.3 × 10^6 um^3/mL) with light intensity of 1000 μmol/m^{2}s and growth temperature of 25°C was used for all experiments. Air was provided at 250 mL/min into well-stirred (500 rpm) vessels. Bacterial contamination was monitored by sampling cultures twice daily and plating on TAP plus 0.4% w/v yeast extract (Bioworld Molecular Life Sciences) agar plates. Cultures grew turbidostatically for two days before performing labeling experiments. The cycling of a peristaltic pump indicated the volume of media added over time to maintain constant turbidity. The time it takes to add a volume of media equivalent to the reactor size (i.e., 500mL) is defined as the doubling time. The media volume added was plotted over time to establish the growth rate.

Sample extraction:

Filtered biomass was extracted at 4°C as described previously (10, 11). Chloroform: methanol (30:70, 3 mL, -20°C) was used to extract biomass from filters at 4°C on a tube rotator (VWR, Radnor, PA) over a 12-hour duration. 150ng of PIPES (piperazine-N, N′-bis[2-ethanesulfonic acid]), norvaline, and ribitol were added to each sample as internal standards. Extracted metabolites and solvent were recovered from the Millipore filter at 4°C, by pushing the extract through a 10ml lure-loc™ syringe (BD, Franklin Lakes, NJ) attached to a Puradisc 25mm 1.0μm GMF filter (Whatman, Marlborough, MA). Water (5mL) was added to induce phase separation, vortexed and centrifuged (5 min, 4000rpms, 4°C) and the aqueous phase was dried in a Centrivap (Labconco, Kansas City, MO) at 27°C, resuspended in 100uL of 50% methanol:water, filtered (4000rpm, 5 min, 0.22μm filter; Corning, Canton, NY), transferred to vials and stored at -80°C.

Measurement of central carbon metabolites in LC-MS/MS:

Metabolite analyses were conducted using a Shimadzu (UFLCXR) HPLC system connected to an AB Sciex triple quadrupole MS equipped with Turbo V™ electrospray ionization (ESI) source using previously described methods (11, 12). Chromatographic separation for sugars, nucleotide sugars and organic acids was achieved using an Infinity Lab Poroshell 120 Z-HILIC column (2.7 μm, 100 x 2.1 mm; Agilent technologies, Santa Clara, CA, USA) upon injecting 5 μL of sample and a gradient elution using solvents A (10 mM ammonium acetate in water, pH 9.0) and B (acetonitrile: 10 mM ammonium acetate, 90:10 v/v, pH 9.0). A flow rate of 0.25 mL/min was used throughout the 20-minute run, utilizing a gradient of 95-70% B over 8 minutes, then to 50% B over the next 4 minutes, followed by a hold at 25% B for 1.5 min. The gradient was then decreased to 30% B over 0.5 min, followed by a hold for 1 min before returning to 95% B for re-equilibrating the column for 6 min. The HPLC eluent was introduced into an electrospray ionization source with the following conditions: ion spray voltage, 4.5 kV (ESI-); ion source temperature, 550°C; source gas 1, 45 psi; source gas 2, 40 psi; curtain gas, 35 psi; and entrance potential, 10. Ions were detected and monitored using a targeted MRM approach as previously described (12). The value for entrance potential was default (-10) for all analytes. For absolute quantification, data were analyzed using the quantitation wizard available in Analyst (v. 1.6.2) software (AB SCIEX, Concord, Canada). Phosphorylated sugars were measured using an Imtakt Intrada Organic Acid column (150 × 2 mm, 3 μm; Kyoto, Japan) following previously described methods and MS parameters (11). Free amino acids were measured in positive ionization mode of mass spectrometry using similar chromatographic and mass spectrometry parameters,

which were used for measuring proteogenic amino acids (see SI Appendix: Supplementary Materials and Methods). Metabolite concentrations were calculated based on calibration curves. Recoveries were assessed using ribitol, PIPES, and Norvaline as internal standards for sugars, sugar phosphates, and amino acids, respectively.

Proteomic Analysis of Protein Levels:

Biomass samples (n=3) for autotrophic and mixotrophic conditions were reduced with 10mM TCEP and alkylated with 25mM iodoacetamide, followed by digestion with trypsin/Lys-C at 37 °C overnight. The digested sample was acidified with 1% TFA, then cleaned up with a C18 pipette tip. The extracted peptides were dried down, and each sample was resuspended in 10 μL 1% acetonitrile/1% formic acid. 5 μL was analyzed by LC-MS using the following method.

LC-MS/MS was carried out on an Orbitrap Fusion Lumos (Thermo Fisher Scientific, San Jose, CA) mass spectrometer coupled with a U3000 RSLCnano HPLC (Thermo Fisher Scientific, San Jose, CA). The peptide separation was carried out on a C18 column (PepMap C18, 75 μm × 50 cm, Thermo Scientific 100 Å, 2.0 μm) at a flow rate of 0.3 μl/min and the following gradient: Time = 0–1 min, 2-8% B; 1-75 min, 8-25% B; 75-88 min, 25-50% B; 88-90 min, 50-90% B; 90-93 min, 90% B isocratic; 93-95 min, 90-2% B; 95-130 min, 2% B isocratic. The mobile phase consisted of A, which had 0.1% formic acid; the mobile phase B had 0.1% formic acid in acetonitrile. Full mass spectra were acquired using the Orbitrap for m/z range 300 to 1800 with a resolution of 60,000 at m/z 400. For full mass spectra, the automatic gain control (AGC) target was set to 200,000 ions with a maximum fill time of 50 ms. Tandem mass spectra were acquired in data-dependent mode using the linear ion trap using Higher-energy Collisional Dissociation (HCD) with a normalized collision energy value of 35. Precursors were isolated using the quadrupole with a window of 1.6 m/z. For tandem mass spectra, the AGC target was set to 10,000 ions with a maximum fill time of 100 milliseconds. Dynamic exclusion was performed with a repeat count of 1, exclusion duration of 15 s, and a minimum MS ion count for triggering MS/MS set to 5000 counts. Precursor selection and subsequent fragmentation had a cycle time of 2 seconds.

Sequence mapping and label-free quantification were done using Proteome Discoverer (version PD 2.4). Briefly, spectra were searched against a Chlamydomonas reinhardtii database with trypsin as the digestion enzyme, carbamidomethylation of cysteine as a fixed modification, and oxidation of methionine and acetylation of the protein N-terminus as variable modifications. Results were then validated against a concatenated decoy database to determine peptide false discovery rates (FDR). Protein quantification was achieved by using the total intensities of all precursors. Proteome Discoverer results were exported as spreadsheets. Significant protein identifications were defined as protein results with at least two unique peptides, and each peptide having a FDR less than 1%. Imputation was performed when two of three replicates had quantified values, and their average was used for the third replicate.

Lipid measurement:

Fatty acid methyl esters (FAME) were quantitated by transmethylation of biomass and quantification with GC-FID as previously described (13). Briefly 5% sulfuric acid containing 0.2% butylated hydroxytoluene (BHT) in methanol, and trihepta- and tripentadecanoin quantification standards was added to biomass (approximately 10 mg dry weight) and heated at 110°C for three hours to create FAMEs for quantification (13, 14). NaCl (0.9% w/v) was added to quench the reaction which was extracted with hexane. FAMEs were quantified on a Thermo FOCUS GC gas chromatography flame ionization detector (GCFID; ThermoFisher Scientific, Waltham, MA) with an Agilent HP Innowax column (30m X .25mm X .25 um) or a Restek DB-23 column (30m X .25mm X .25 um). The operating parameters for the GC-FID included injection of two microliter samples at a 15:1 split. The inlet temperature was maintained at 250°C and the carrier gas set to 1.2 microliters/second. The oven temperature was set to 180°C for one minute then ramped at 20°C/min to 260°C where the temperature was held for seven minutes. Independent quantification of a known amount of stearic acid (C18:0) served as a positive control.

Starch measurement:

Biomass (approximately10 mg DW) was washed twice with 80% ethanol to remove free sugars then treated with DMSO at 110°C for 10 minutes. Starch was enzymatically digested and quantified spectrometrically as detailed in the Megazyme Total Starch Assay kit (Megazyme™ International, Ireland) and described elsewhere (3) using AOAC Official Method 996.11, modified for 96-well plate use. Briefly, pellets containing starch were heated to 110°C for 12 minutes (vortexing every 4 min) in the presence of a heat-stable α-amylase followed by the addition of amyloglucosidase at 50°C for 1 hour. After centrifugation, serial dilutions of the supernatant were incubated with commercially supplied GOPOD reagent for 20 min prior to measurement of absorbance at 510 nm. Corn starch and glucose were used as controls and to establish the efficiency of the process.

Proteinogenic amino acid measurement:

Biomass (approximately 10 mg DW) was hydrolyzed in 250 microliter insert vials for protein quantification. 10 μM labeled amino acid mix (MSK-CAA-1, Cambridge Isotope Laboratories) was added as internal standard to gauge losses during hydrolysis and quantification. Samples were vapor hydrolyzed following the method previously described (15) using 4M Methane sulfonic acid with 0.2% tryptamine (w/v) in a vacuum sealed Kimball® hydrolysis chamber (Vineland, New Jersey, Part #896820) for 24 hours at 110°C. Samples were then dried in a speed vac centrifuge and resuspended in 200 microliters of 50 % MeOH and quantified using LC-MS/MS following the method previously described (15). Briefly, hydrolyzed amino acids were injected on an Infinity Lab Poroshell 120 Z-HILIC column (2.7 µm, 100 x 2.1 mm; Agilent technologies, Santa Clara, CA, USA) with positive ion mode and metabolites preferentially eluted using acetonitrile: 20 mM ammonium formate (90:10 v/v) (A) and 20 mM ammonium formate in water (B). Both buffers were adjusted to pH 3.0. A flow rate of 0.25 ml/min was used with a gradient of 95 to 60% B over 4.5 minutes, 60 to 25% B over the next 1.5 min, a hold at 25% B for 1 min, a return to 95% B over 1.75 min, and a final re-equilibration for 5.25 min. Electrospray ionization parameters included: ion spray voltage set to 4.5 kV, ion source temperature to 400°C, source gas 1 to 40 psi, source gas 2 to 30 psi, curtain gas to 35 psi, and entrance potential to 10 V. Ions were detected and monitored using a targeted MRMs based on direct injections of individual amino acid standards. Amino acid concentrations and recoveries were calculated based on direct comparison of peak areas with their respective labeled internal standards through an isotopic dilution approach (15).

Nucleotide calculation:

Nucleotide flux was approximated using the reported amount of DNA per cell ($1.3x10^{-13}$ g), and a DNA:RNA ratio of 1:28 for *C. rienhardtii* (16) to establish the total amount of nucleotide per cell ($3.77x10^{-12}$g). Cell number was correlated with optical density at 750nm resulting in an estimate of the number of cells at a reading of 0.6 (optical density of photobioreactor cultures). $4.09x10^{-4}$ g of nucleotides in a 50mL culture at an optical density of 0.6 resulted in micromolar levels of purines and pyrimidines presuming equivalent levels of each nucleotide. Milligrams of biomass expected from 50mL of culture was plotted against optical density to determine the expected amount of biomass at an OD750 of 0.6. This constant was used to determine the µmol of purines and pyrimidines per gram of biomass. The doubling rate of TP and TAP cultures were used to establish biomass flux estimates for nucleotides in mixotrophic and autotrophic growth conditions.

RNA Preparation:

For each strain, RNA was extracted from 50 mL of a TAP or TP-grown culture (total cell biomass volume of 180 to 190 × 10^6 µm^3 per ml). Replicates were collected from bioreactors after approximately two culture doublings. Cells were centrifuged in 0.005% (w/v) Tween-20 (3500 RCF for 10 min), and the pellet was resuspended in RNase-free water to a volume of 250 µL. An equal volume of 2x lysis buffer (100 mM Tris-HCl pH 8, 400 mM NaCl, 40 mM EDTA pH 8, 4% SDS, 2 mg/ml Proteinase K) pre-warmed at 50 ºC, was added by dripping the cell suspension into the lysis buffer while gently vortexing. The cell lysate was placed

on an orbital shaker at low speed for 20 min at room temperature. Five mL of TRIzol was added to the lysate, vortexed, flash frozen in liquid nitrogen, and stored at -80 ºC until use. The TRIzol/lysate mixture was thawed at room temperature for RNA extraction and transferred to 15 mL MaXtract High Density tubes (Qiagen #129065). One mL of chloroform was added and shaken vigorously. Tubes were incubated for 5 min at room temperature and centrifuged at 1500 RCF for 5 min. The aqueous phase was then poured into a fresh tube and combined with 2.5 mL of 2-propanol by gently inverting and incubated for 10 min at room temperature. The mixture was aliquoted into smaller tubes and spun at 15000 RCF for 10 min at 4 ºC. The pellet was washed with 75% ethanol, dried briefly, and resuspended in water. In-solution DNase treatment was performed according to the manufacturer's instructions (RNase-Free DNase set, Qiagen #79254). RNA concentration was measured by a Pearl nanophotometer (Implen), and the quality of RNA was assessed by agarose gel electrophoresis.

DNA Library Preparation and Sequencing:

Library preparation and sequencing with RNA samples was performed by Novogene Corporation Inc. After passing quality control requirements, mRNA was enriched using oligo(dT) beads and randomly fragmented. cDNA was synthesized using mRNA as a template with random hexamer primers. Second-strand synthesis was initiated using a custom second-strand synthesis buffer (Illumina), dNTPs, RNase H, and DNA polymerase I. After terminal repair, A tailing and sequencing adaptor ligation, the double-stranded cDNA library was completed through size selection and PCR enrichment and subjected to Illumina paired-end sequencing that extended 150 bases.

Transcriptome Sequencing and Differential Expression Analysis:

The quality of sequenced reads was first assessed using FastQC version 0.11.9 (https://www.bioinformatics.babraham.ac.uk/projects/fastqc/). Reads were trimmed using Trimmomatic (17) in the paired-end mode. Adaptor sequences (TruSeq3-PE.fa) and leading and trailing bases (with scores below 3, deemed as low quality) were removed. Reads were scanned using a four-base sliding window and trimmed when the average quality score dropped below 20. Trimmed reads shorter than 20 nt were discarded. Paired trimmed reads were used for mapping and were aligned to the Chlamydomonas genome assembly (Creinhardtii_281_v5.0.fa, https://phytozome.jgi.doe.gov) using STAR (18), with the following settings: --outFilterMultimapNmax 1 and --outFilterMismatchNmax 0. Prior to mapping, the genome index was created using the Creinhardtii_281_v5.5.gene_exons.gff3 file (https://phytozome.jgi.doe.gov). Between 26 and 30 million uniquely-mapped reads per sample were assigned to 17,741 gene models described in Creinhardtii_281_v5.5.gene_exons.gff3 using HTSeq-count (--stranded=no --nonunique=none --mode=union). For input into HTSeq, the gff3 file was reformatted to remove carriage returns and to add the v5.5 Gene ID to the end of each exon line. Three transcripts (Cre02.g102302, Cre10.g456460, Cre16.g666451) in the original gff3 had IDs that did not match their parent ID. These were manually changed to Cre02.g102276, Cre10.g456420, Cre16.g666334, respectively, to match the parent ID. Raw read counts obtained from HTSeq were used as input for differential gene expression analysis using DESeq2 (19) (DESeq2 version 1.24.0, R version 3.6.0). Genes having at least one read in at least one replicate were kept in the DESeq input. In addition, FPKM (fragments per kilobase per million mapped reads) normalized expression values were also calculated after adding one read count to each gene. Genes that had a basal FPKM value of >= 1.0 in at least one sample were kept in the analysis. TAP upregulated genes were then obtained using a TAP/TP log2 fold change cutoff of >= 1.0 and false discovery rate (FDR) < 0.05. TP upregulated genes were obtained using a TAP/TP log2 fold change cutoff of <= -1.0 and FDR < 0.05.

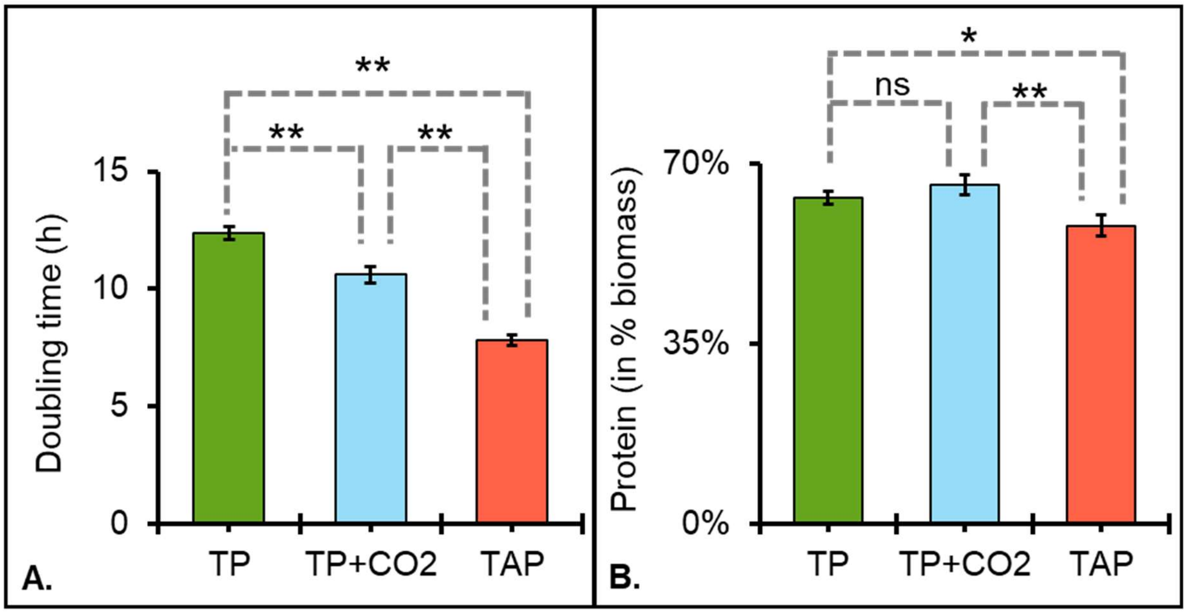

Figure S1. Differences in doubling time (A) and protein content (B) of cultures under three growth conditions. A 350 mL steady-state photobioreactor cultures were grown mixotrophically (TAP, orange bars), autotrophically with air (TP, 400 ppm CO_2, green bars), and autotrophically with supplemental CO_2 (TP+CO_2, 10,000 ppm CO_2, blue bars). Results in each bar graph were compared using a one-way ANOVA test, with Tukey's HSD test applied for post-hoc pairwise comparisons. Statistical significance is indicated by * p<.01; ** p<.0001; and ns (not significant). Doubling time data are presented as mean ± S.D. (n= 48-74). Proteinogenic biomass data are presented as mean ± S.D. (n=5).

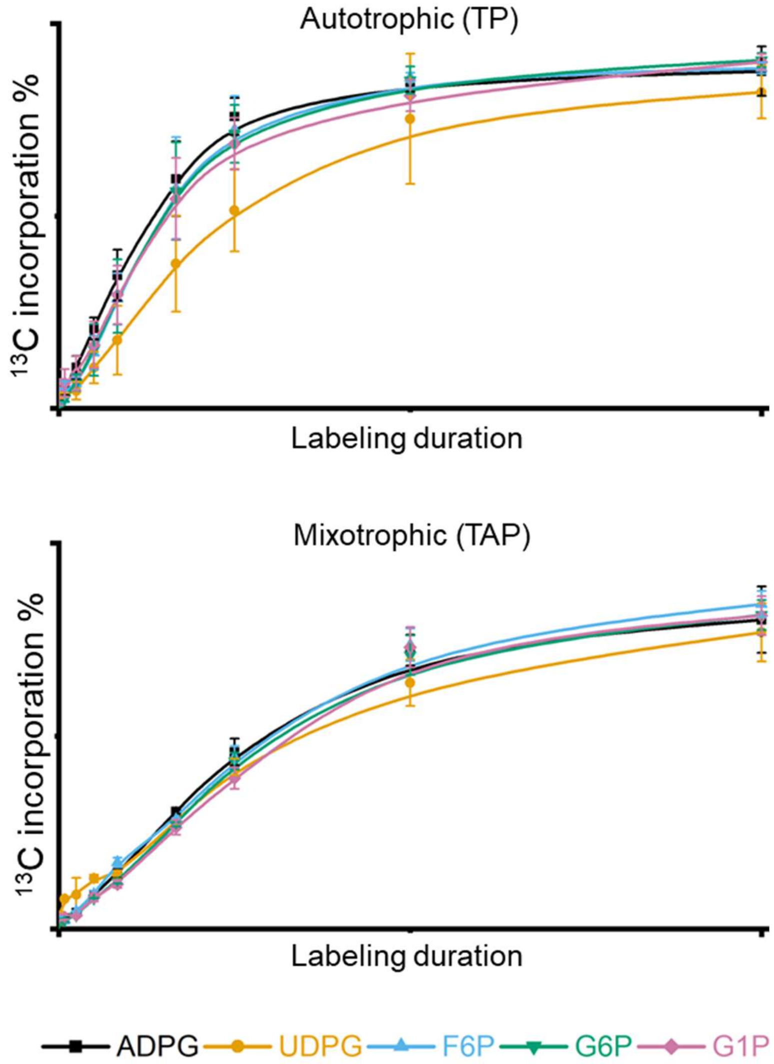

Figure S2. Carbohydrate metabolism in Chlamydomonas. Similar labeling in ADPG, UDPG, F6P, G6P and G1P in both autotrophic and mixotrophic conditions. ADPG: adenosine diphosphate glucose; UDPG: uridine diphosphate glucose; F6P: fructose 6-phosphate; G6P: glucose 6-phosphate; G1P: glucose 1-phosphate. Mean ± S.D.; n=4 for TP and n=6 for TAP.

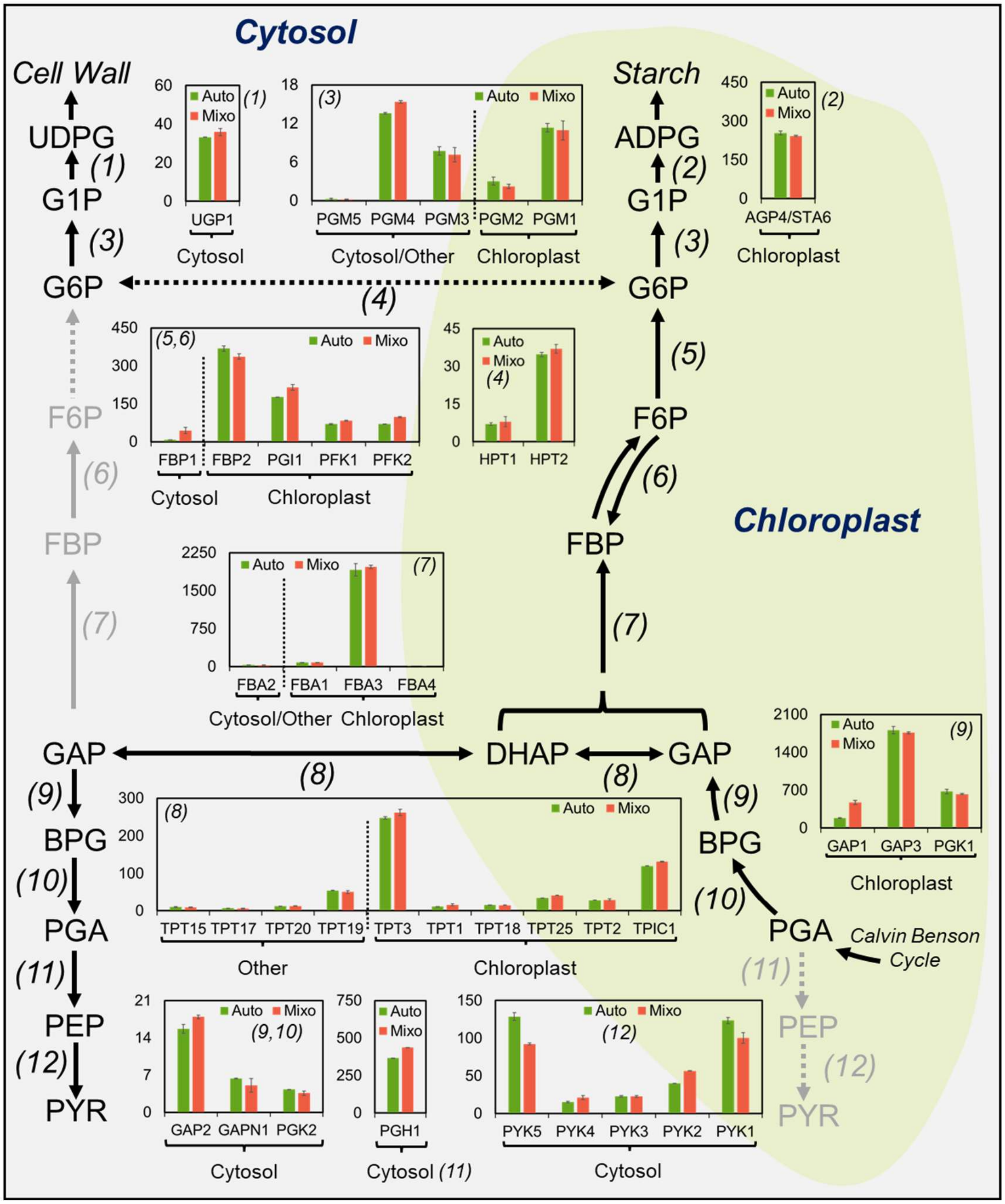

Figure S3. Expression level of key transcripts in central metabolism. Gene expression was absent from steps to convert triose phosphate to hexose phosphate in the cytosol. Other gene levels were comparable between mixotrophic and autotrophic conditions. Enzymes: (1) UDP-glucose pyrophosphorylase, (2) ADP-glucose pyrophosphorylase, (3) phosphoglucomutase, (4) hexose-phosphate transporter, (5) phosphoglucose isomerase, (6) phosphofructokinase / fructose-bisphosphatase, (7) fructose-bisphosphate aldolase, (8) triose-phosphate transporter / triose phosphate isomerase, (9) GAP dehydrogenase, (10) phosphoglycerate kinase, (11) phosphopyruvate hydratase (enolase), and (12) pyruvate kinase. All other abbreviations used are listed in Dataset S1.

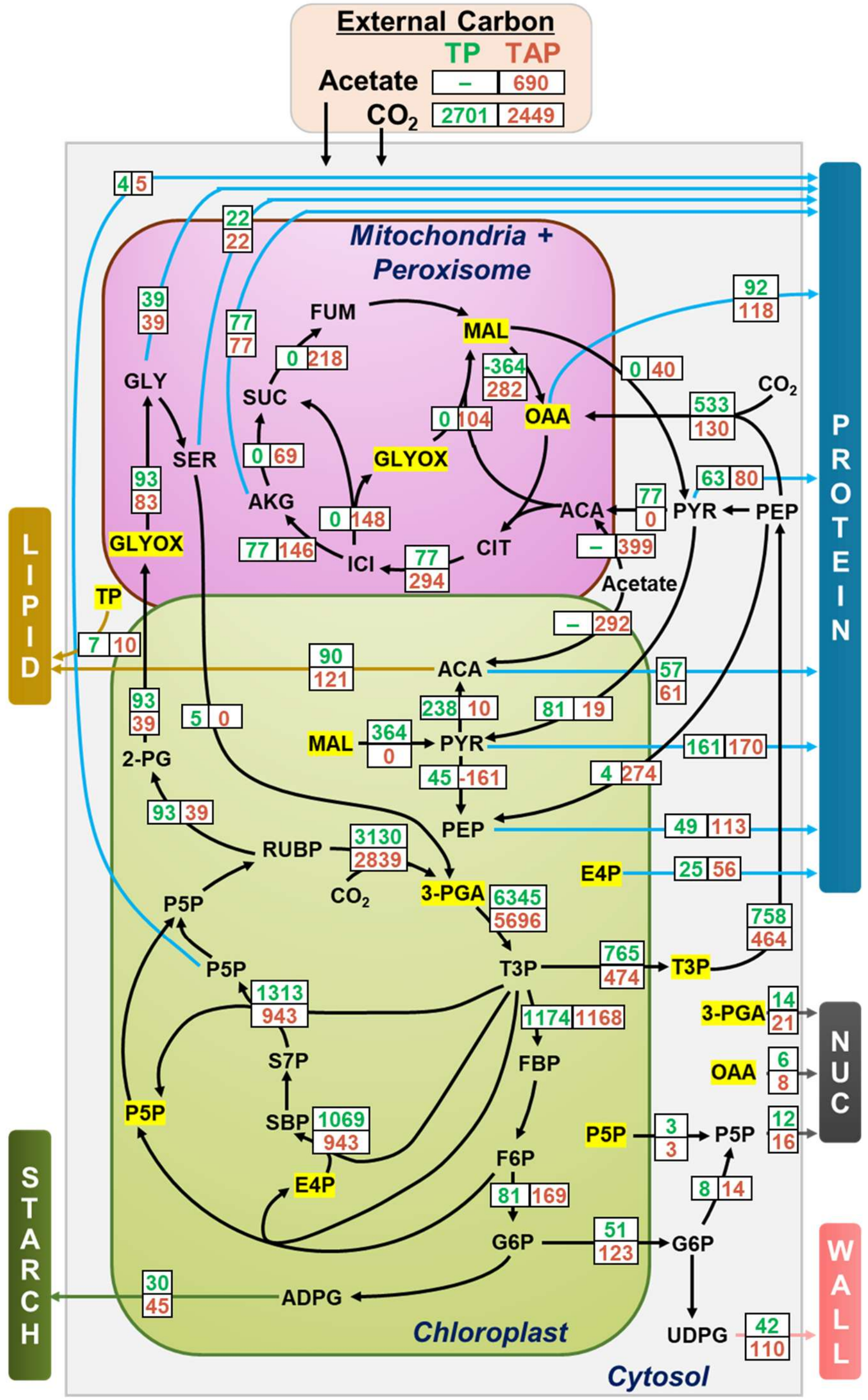

Figure S4. Metabolic flux maps of autotrophic and mixotrophic Chlamydomonas. This figure is related to Figure 2. Net flux values determined from TP (green) and TAP (red) conditions are the medians of the 95% flux confidence intervals and are presented in units of µmol*g^{-1}(DW)*h^{-1}. These flux values are rescaled to doubling time differences in the Figure 2. The calculation is presented in Dataset S8. Metabolites highlighted in yellow are visualized multiple times in different locations on the map but were considered as a single pool in the flux analysis. The breakdown of protein and starch was included in the flux analysis (Dataset S8) but is not presented here to simplify the flux map. All abbreviations used are listed in Dataset S1.

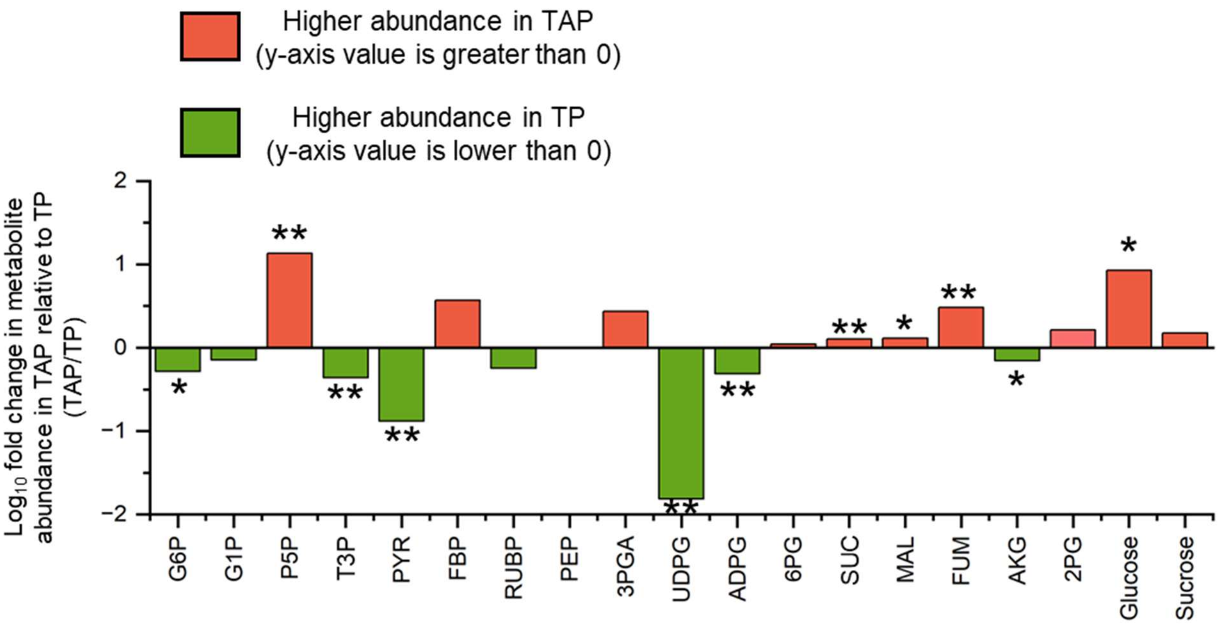

Figure S5. Difference in metabolite abundance of sugars, phosphorylated and nucleotide sugars and organic acids between autotrophic and mixotrophic Chlamydomonas. Data are presented as log10 fold change; n=5. * p=0.01 to <0.05; ** p<0.01. All abbreviations used are listed in Dataset S1.

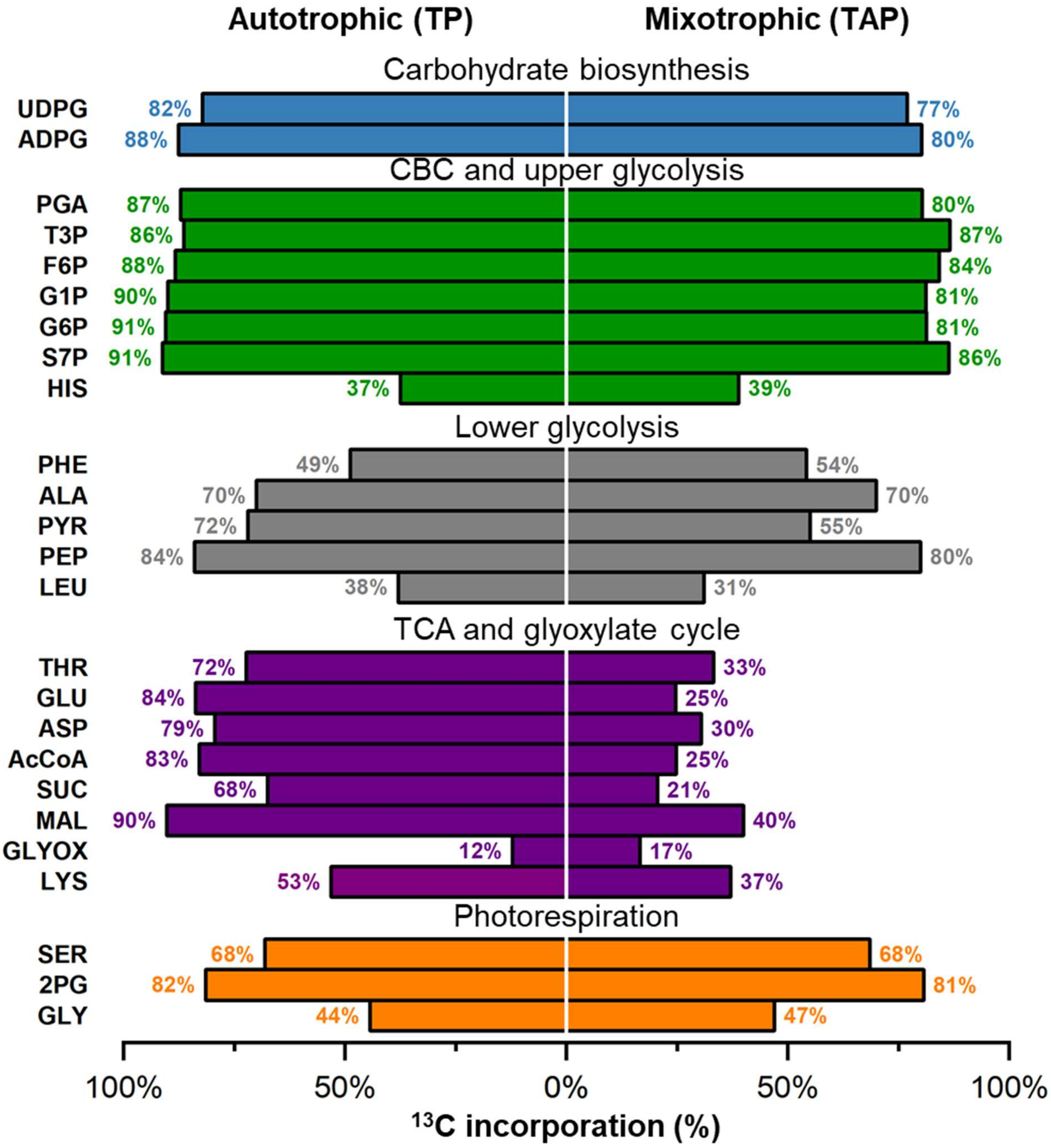

Figure S6. Labeling differences between autotrophic and mixotrophic chlamy. (A) Comparison of average isotope incorporation in central metabolism and amino acids at 1 h. Mean (n=4 for TP and n=6 for TAP) is presented. All abbreviations used are listed in Dataset S1.

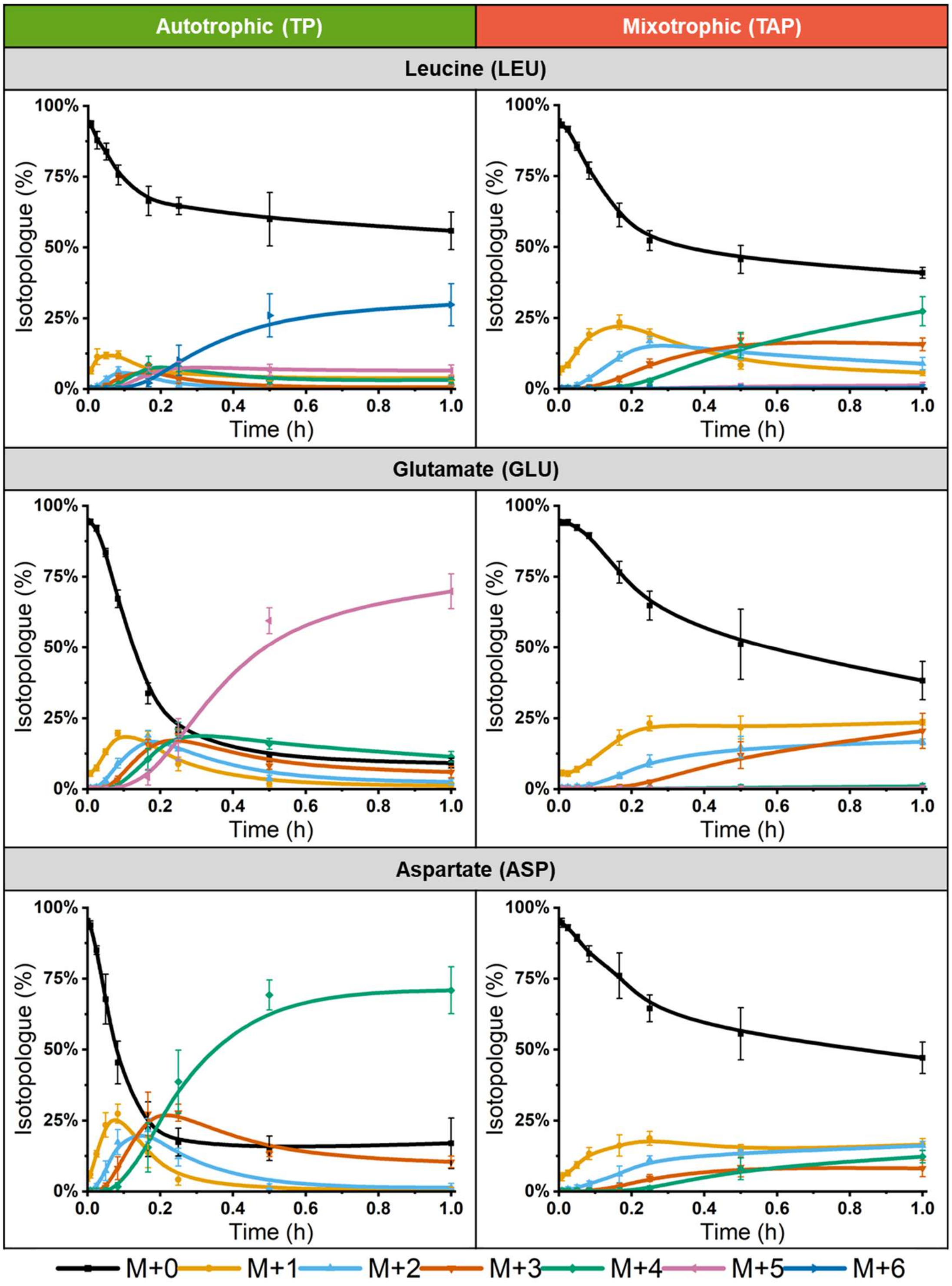

Figure S7. Labeling differences in glutamate, aspartate and leucine between autotrophic and mixotrophic Chlamydomonas. Due to the presence of unlabeled acetate (a 2-carbon containing compound), the two heaviest isotopologues were absent or present at reduced levels in mixotroph; e.g., (M+4) and (M+5) of glutamate, (M+3) and (M+4) of aspartate, and (M+5) and (M+6) of leucine. Data points are Mean ± S.D.; n=4 for TP and n=6 for TAP, lines drawn to show clear trends.

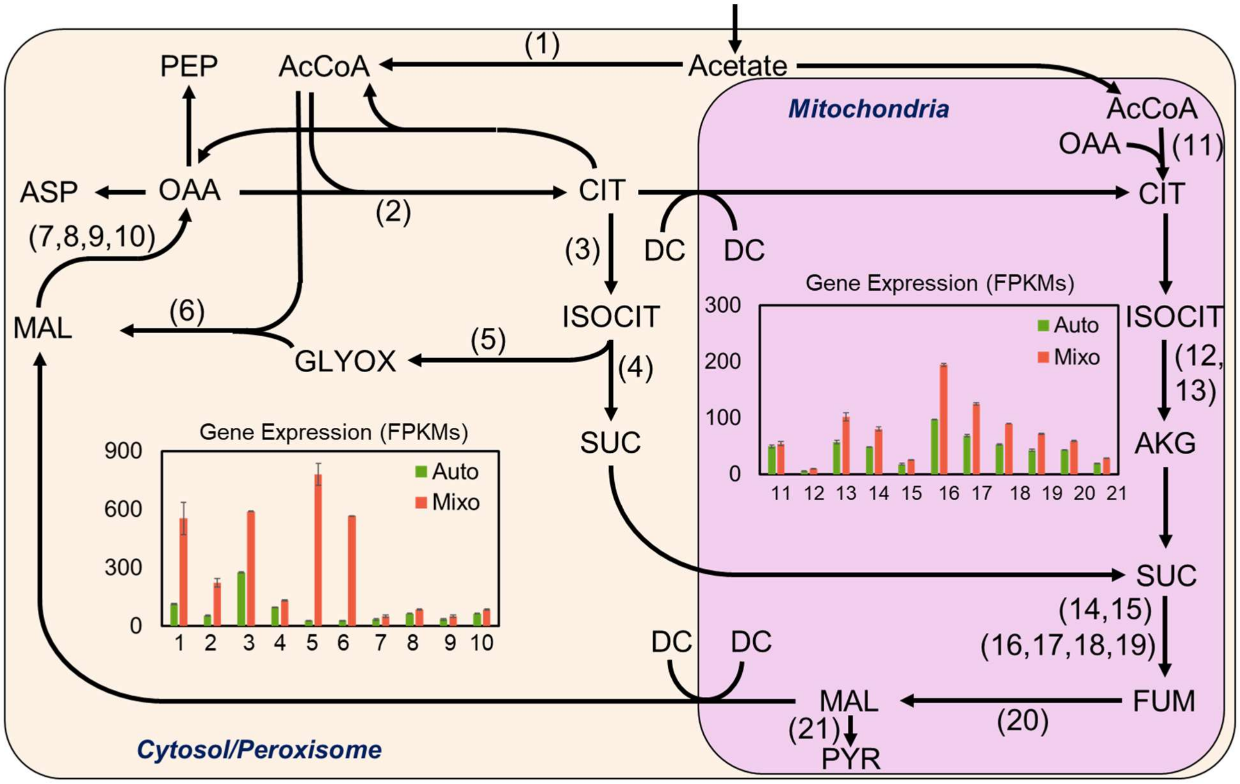

Figure S8. Differential expression of transcripts supports glyoxylate cycle in mixotrophic metabolism. RNAseq gene expression in genes associated with glyoxylate and the TCA cycle. Enzymes: (1) acetyl-CoA synthetase; (2) citrate synthase; (3) aconitase; (4) isocitrate dehydrogenase; (5) isocitrate lyase; (6) malate synthase; (7-10) malate dehydrogenase; (11) mitochondrial citrate synthase; (12,13) isocitrate dehydrogenase; (14,15) succinyl-CoA synthetase; (16-19) succinate dehydrogenase; (20) fumarase; (21) mitochondrial malic enzyme. All abbreviations used are listed in Dataset S1.

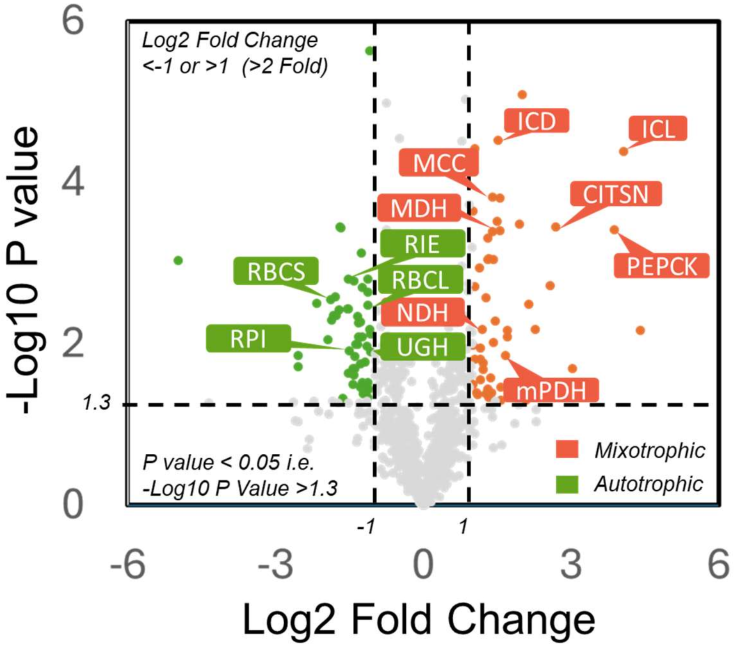

Figure S9. Proteomics indicates autotrophic assimilation and mixotrophic mitochondrial metabolism. Analysis of proteins indicated preferentially elevated levels of central metabolic genes related to carbon assimilation for autotrophic conditions and tricarboxylic acid cycle and electron transport in the mitochondria for mixotrophic conditions, autotrophic, n=3; mixotrophic, n=3 (Dataset S3). A number of proteins were measured in only one of the two conditions; thus log FC and p-Value statistics do not apply, and difference is very significant including: acetyl-CoA synthetase and acetate kinase involved in activation of acetate, malate synthase that is a signature for glyoxylate cycle activity that favored TAP. For TP, Rubisco and sugar phosphate steps are presented, ribosomal and photosynthetic proteins that were also significant were numerous and are not outlined (Dataset S3). Abbreviations: CITSYN, citrate synthase; ICD, isocitrate dehydrogenase; ICL, isocitrate lyase; MCC, mitochondrial cytochrome c oxidase; MDH, malate dehydrogenase; NDH, mitochondrial NADH dehydrogenase; PEPCK, phosphoenolpyruvate carboxykinase; mPDH, mitochondrial pyruvate dehydrogenase; RIE, Rieske/ferrodoxin protein; RBCL, ribulose bisphosphate carboxylase large chain; RBCS, ribulose bisphosphate carboxylase small chain; RPI, ribose 5-phosphate isomerase; UGDH, UDP-glucose 6-dehydrogenase).

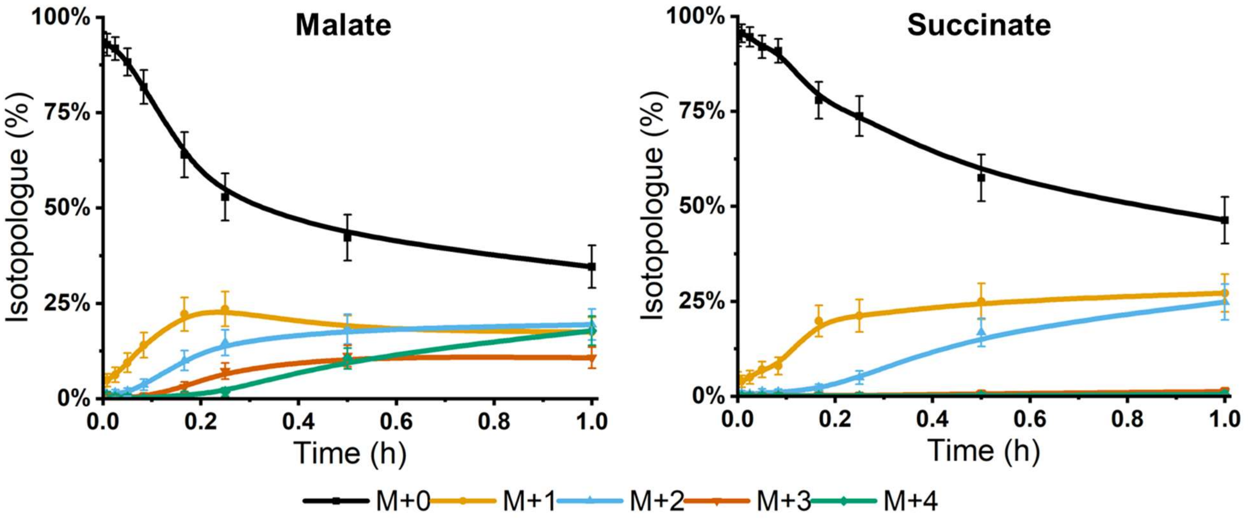

Figure S10. Difference in isotopologue distribution between malate and succinate in mixotrophic condition. Because unlabeled acetate (a 2-carbon compound) was present, the heaviest succinate isotopologues (M+4 and M+3) were not detected. In contrast, these isotopologues appeared at reduced levels in malate, reflecting additional contributions from PEPC activity, which introduces photosynthetically fixed labeled carbons into the TCA cycle. Data are presented as mean ± S.D. (n=6); lines are included to highlight trends.

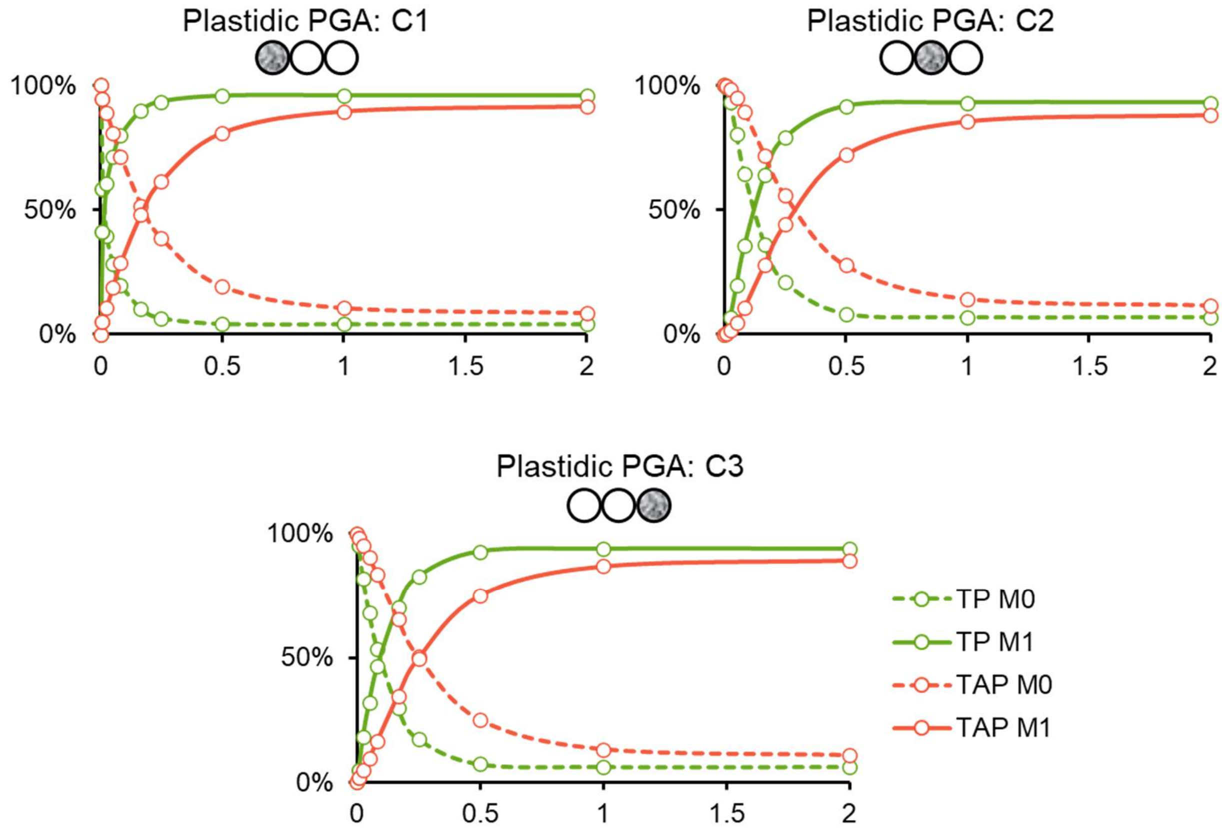

Figure S11. Labeling difference in all three carbons of plastidic PGA between TP and TAP. Positional carbon enrichment was simulated from the flux maps. TP and TAP media describe autotrophic and mixotrophic conditions, respectively. PGA: 3-phosphoglycerate.

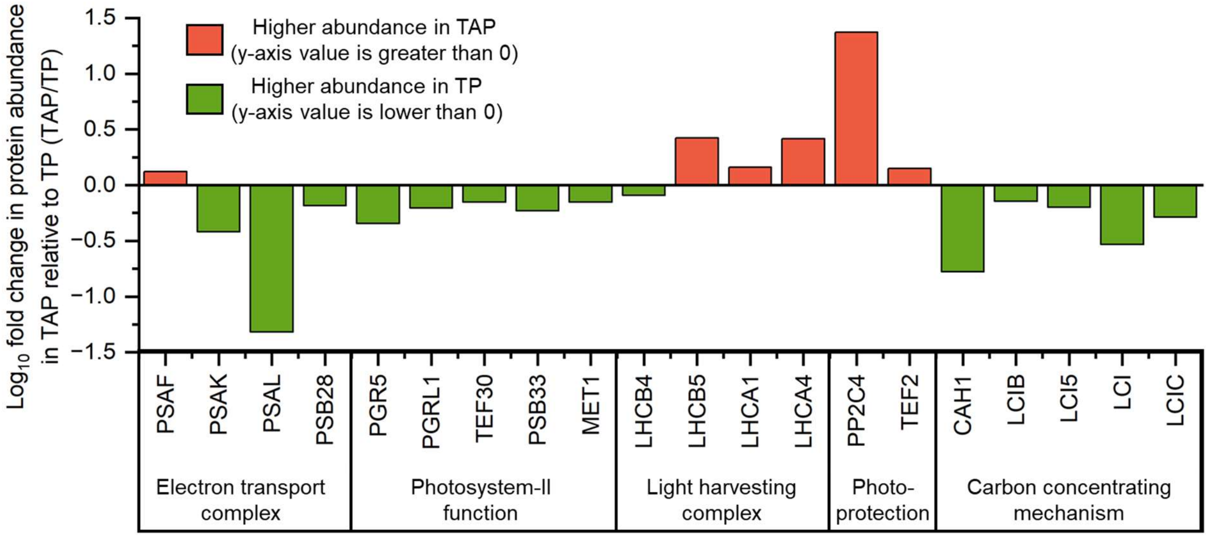

Figure S12. Differences in protein abundances related to oxygenic photosynthesis and carbon concentrating mechanism components in autotrophic and mixotrophic Chlamydomonas. Data are presented as log10 fold change; n=3.

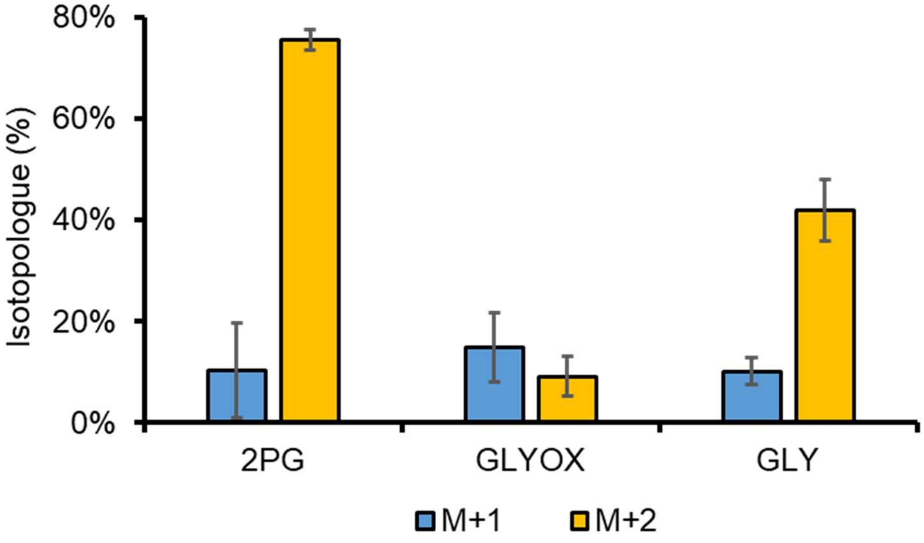

Figure S13. Distribution of labeled isotopologues in 2PG, GLYOX and GLY in mixotrophic condition. At near isotopic steady state (1h), the ratio of M+2 and M+1 in 2PG, GLYOX and GLY is 7.4, 0.6 and 4.2. Higher M+1 than M+2 in GLYOX, which was the opposite of 2PG, indicated that GLYOX production was influenced by unlabeled acetate through glyoxylate cycle. Labeling in GLY fell between 2PG and GLYOX, which indicated that glyoxylate cycle contributed to glycine production via glyoxylate. 2PG: 2-phosphoglycolate; GLYOX: glyoxylate; GLY: glycine. Mean ± S.D.; n=6.

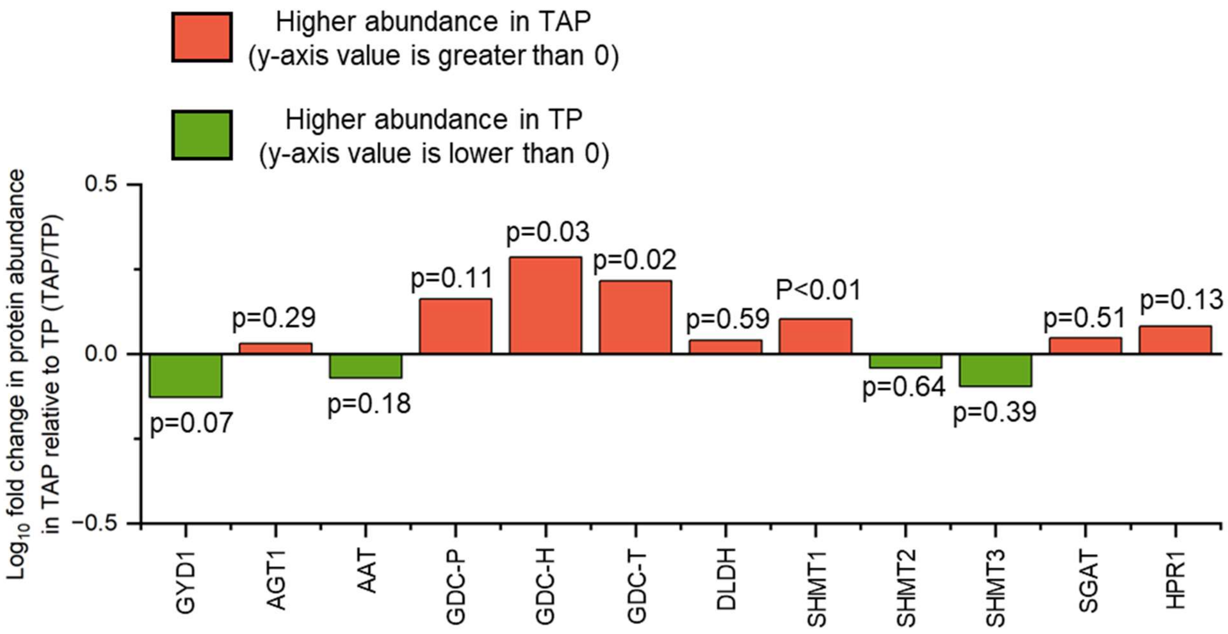

Figure S14. Difference in photorespiratory protein abundances between autotrophic and mixotrophic Chlamydomonas. Data are presented as log10 fold change; n=3.

Datasets (separate file):

S1: Abbreviation used in the manuscript

S2: Complete list of reactions and atom transitions for *C. reinhardtii* metabolic network in TP and TAP conditions

S3: Proteomics measurement in TP and TAP

S4: Transcriptomics measurement in TP and TAP

S5: Measured biomass of protein, starch and lipid used in the ^{13}C INST-MFA models

S6: Calculated biomass of nucleosides and cell wall used in the ^{13}C INST-MFA models

S7: Measured isotopologue abundances of metabolites used in the ^{13}C INST-MFA models

S8: Net and exchange fluxes determined by ^{13}C INST-MFA and flux comparison between TP and TAP

S9: ATP production from TCA cycle in TP and TAP

SI references:

1. D. K. Allen, P. D. Bates, H. Tjellström, Tracking the metabolic pulse of plant lipid production with isotopic labeling and flux analyses: Past, present and future. *Progress in Lipid Research* **58**, 97–120 (2015).

2. D. K. Allen, Assessing compartmentalized flux in lipid metabolism with isotopes. *Biochimica et Biophysica Acta (BBA) - Molecular and Cell Biology of Lipids* **1861**, 1226–1242 (2016).

3. K. L. Chu, *et al.*, Metabolic flux analysis of the non-transitory starch tradeoff for lipid production in mature tobacco leaves. *Metabolic Engineering* **69**, 231–248 (2022).

4. F. Ma, L. J. Jazmin, J. D. Young, D. K. Allen, Isotopically nonstationary 13C flux analysis of changes in Arabidopsis thaliana leaf metabolism due to high light acclimation. *Proc. Natl. Acad. Sci. U.S.A.* **111**, 16967–16972 (2014).

5. Y. Xu, X. Fu, T. D. Sharkey, Y. Shachar-Hill, and B. J. Walker, The metabolic origins of non-photorespiratory CO2 release during photosynthesis: a metabolic flux analysis. *Plant Physiology* **186**, 297–314 (2021).

6. X. Johnson, J. Alric, Central Carbon Metabolism and Electron Transport in Chlamydomonas reinhardtii: Metabolic Constraints for Carbon Partitioning between Oil and Starch. *Eukaryot Cell* **12**, 776–793 (2013).

7. M. Bockwoldt, I. Heiland, K. Fischer, The evolution of the plastid phosphate translocator family. *Planta* **250**, 245–261 (2019).

8. W. Huang (黄伟超), *et al.*, Chlamydomonas mutants lacking chloroplast TRIOSE PHOSPHATE TRANSPORTER3 are metabolically compromised and light sensitive. *The Plant Cell* **35**, 2592–2614 (2023).

9. E. H. Harris, D. B. Stern, G. B. Witman, Eds., "Chlamydomonas in the Laboratory" in *The Chlamydomonas Sourcebook (Second Edition)*, (Academic Press, 2009), pp. 241–302.

10. F. Ma, L. J. Jazmin, J. D. Young, D. K. Allen, Isotopically Nonstationary Metabolic Flux Analysis (INST-MFA) of Photosynthesis and Photorespiration in Plants. *Methods Mol Biol* **1653**, 167–194 (2017).

11. S. Koley, K. L. Chu, S. S. Gill, D. K. Allen, An efficient LC-MS method for isomer separation and detection of sugars, phosphorylated sugars, and organic acids. *Journal of Experimental Botany* **73**, 2938–2952 (2022).

12. J. J. Czajka, S. Kambhampati, Y. J. Tang, Y. Wang, D. K. Allen, Application of Stable Isotope Tracing to Elucidate Metabolic Dynamics During Yarrowia lipolytica α-Ionone Fermentation. *iScience* **23**, 100854 (2020).

13. D. K. Allen, J. D. Young, Carbon and Nitrogen Provisions Alter the Metabolic Flux in Developing Soybean Embryos. *Plant Physiology* **161**, 1458–1475 (2013).

14. Y. Li-Beisson, *et al.*, Acyl-Lipid Metabolism. *Arabidopsis Book* **11**, e0161 (2013).

15. S. Kambhampati, J. Li, B. S. Evans, D. K. Allen, Accurate and efficient amino acid analysis for protein quantification using hydrophilic interaction chromatography coupled tandem mass spectrometry. *Plant Methods* **15**, 46 (2019).

16. A. M. J. Kliphuis, *et al.*, Metabolic modeling of Chlamydomonas reinhardtii: energy requirements for photoautotrophic growth and maintenance. *J Appl Phycol* **24**, 253–266 (2012).

17. A. M. Bolger, M. Lohse, B. Usadel, Trimmomatic: a flexible trimmer for Illumina sequence data. *Bioinformatics* **30**, 2114–2120 (2014).

18. A. Dobin, *et al.*, STAR: ultrafast universal RNA-seq aligner. *Bioinformatics* **29**, 15–21 (2013).

19. M. I. Love, W. Huber, S. Anders, Moderated estimation of fold change and dispersion for RNA-seq data with DESeq2. *Genome Biology* **15**, 550 (2014).
